# Talca no es Santiago: Moral geography, rurality, and infrastructure of possibility in LGBTIQA+ violence and survival

**DOI:** 10.1371/journal.pone.0360622

**Published:** 2026-10-14

**Authors:** Wilson Orlando Albornoz Fuentes, Cecilia Del Pilar Valenzuela Gajardo, Thiare Anaís Cid Ramírez

**Affiliations:** Universidad Autónoma de Chile, Talca, Chile; Universidad de Chile, CHILE

## Abstract

**Background:**

Violence against LGBTIQA+ individuals is not limited to discrete acts of aggression. It also constitutes a territorial ecology that unevenly distributes habitability, safety, and recognition. This study examines how the statement “Talca is not Santiago” works as more than a comment on city size: it names a moral geography where reputation, social surveillance, and uneven access to infrastructure produce distinct thresholds of visibility, risk, and agency.

**Methods:**

We used Reflexive Thematic Analysis (RTA) following Braun and Clarke to analyze a subcorpus of seven individual interviews and one focus group (n = 7) conducted in Talca and one focus group (n = 5) conducted in Santiago, drawn from a broader research project according to predefined criteria (density of territorial references and representation of both study sites). Reporting follows COREQ guidelines (S1 Checklist). We recruited participants through LGBTIQA+ organizations and social networks; participants self-identified with non-hegemonic sexual-gender identities, were over 18, and reported subjective experiences of violence. The analytical sample is composed predominantly of young (19–35 years) university-educated participants, with representation of cisgender, transmasculine, and non-binary identities and no trans women. We calibrate the scope of our conclusions accordingly.

**Results:**

We constructed three themes: (1) Infrastructure of possibility—LGBTIQA+ life becomes practicable or precarious depending on available circuits of information, networks, and spaces; (2) Surveillance and reputation—in medium-sized cities and rural environments, social legibility intensifies daily control and shrinks the margin to maneuver, which also affects the possibility of leaving violent relationships; (3) Mobility and education as partial territorial rupture—moving for studies and traveling through urban circuits reconfigures future horizons without erasing access asymmetries.

**Conclusions:**

Within the scope of a young, predominantly cisgender, university-educated sample, territorial scale operates as a constitutive condition of violence and survival, not as a backdrop. Interventions on LGBTIQA+ violence therefore need to address territorial conditions: connectivity, information circulation, community spaces, and confidentiality safeguards adapted to medium-sized cities and ruralities.

## 1. Introduction

Violence against persons and bodies that exceed heterocisnormative mandates is not only a matter of discrete acts of aggression. It also operates as a social and territorial structure: a set of material, relational, and symbolic conditions that unevenly distributes habitability, safety, and recognition [1]. From this perspective, daily space does not work as a passive stage on which events unfold, but as an active condition that organizes which forms of visibility become tolerable, which become punishable, and which survival strategies become thinkable at a given urban scale [2–6]. Sociology [7], psychology [8], anthropology [9], and gender studies [10] have argued that geographical distribution does not simply index city size or density: it indexes how territory, as a social production of space, distributes anonymity, access, information circuits, and local grammars of reputation. Territory thereby configures a regime of legibility that defines which forms of appearing are intelligible, which behaviors are sanctionable, and which margins of agency become practicable for sexual and gender dissidence. In this sense, territory does not only “expose” or “protect”; it produces ways of being and conducting oneself in daily life [11].

In gender studies, and particularly LGBTIQA+ studies, scholars have argued that visibility is an ambivalent political category [12]. Visibility can enable recognition and collective articulation, but it can also expose and harm when normative regimes capture public legibility to sanction difference. This becomes especially clear when hyperexposure normalizes conduct and rumor disciplines transgression [13–15]. Much of this debate, however, treats visibility as if it were an individual attribute or a personal decision (“coming out” or not, “showing oneself” or “hiding”). This framing underestimates how visibility is produced in unequal territorial infrastructures, where city scale, network density, the availability of spaces, and the circulation of information set differential conditions for appearing without immediately becoming capturable by social sanction [4,16,17]. In medium-sized cities and rural contexts, where relational density intensifies the legibility of body and bond, visibility ceases to be a mere expressive option and becomes a territorial calculation of self-conduct: the daily work of adjusting gestures, affects, and presences. Its costs are paid not only in public space but in the intimate fabric of family, neighborhood, school, work, and extended networks [9,18].

In Latin America, experiences of violence and survival are territorialized in situated circuits of sociability, information, and mobility. Access to support networks, community scenes, and repertoires of meaning is not distributed homogeneously between those who inhabit a medium-sized city in the center-south and those who circulate through the metropolis [1,7]. Read this way, territorial difference can become a difference in future: some spaces expand horizons of biographical continuity, and others restrict existence to forms of discretion, affective self-censorship, or forced permanence in small networks where “leaving” a relationship or a conflict may mean losing the only available circuit [4,18,19]. Under this logic, territorial inequality does more than modulate the risk of external aggression. It reconfigures how intracommunity violence, when it emerges, can be faced, narrated, resisted, or silenced, because the chance to recompose networks, find other spaces, or hold on to anonymity depends on a spatial infrastructure that is not guaranteed for everyone.

The Chilean case is analytically relevant for two reasons. First, Chile’s geographical and demographic distribution configures urban scales and gradients of rurality and city with unequal access to networks, services, information, and spaces of sociability [7,20]. Second, the literature reports that the validation and reproduction of heterocisnormative and masculinizing discourses and practices is not homogeneous: it varies with territorial configurations and local normative climates, producing differentiated thresholds of visibility, risk, and agency [8,9]. Two main gaps persist. The first is theoretical: much research has conceptualized violence against non-hegemonic subjects mainly from the metropolitan experience, with a recurrent emphasis on Santiago, because of the concentration of research resources and the greater visibility of LGBTIQA+ organizations in the capital [7]. This tends to universalize metropolitan dynamics and to underestimate how territory produces specific conditions of subjectivation, moral control, and access to care infrastructures. The second gap is methodological: studies that intersect the influence of rural contexts and medium-sized cities with the situated reproduction of violence against feminized corporalities and LGBTIQA+ persons remain scarce, as do studies on the concrete possibilities of asking for help, recomposing networks, and sustaining survival without reputational exposure [1,8].

The objective of this article is therefore to analyze how LGBTIQA+ persons construct and narrate territorial difference in two Chilean contexts—Talca and Santiago—and along the rurality-city gradient. We treat these as geographies where access to information, the availability of networks and spaces, and local economies of reputation work as conditions of violence and, at the same time, as conditions of LGBTIQA+ survival.

In brief, the analysis constructed three themes. First, LGBTIQA+ life becomes practicable or precarious according to an infrastructure of possibility: the information, recognizable support references, and spaces available at a given urban scale. Second, in the medium-sized city and its adjacent ruralities, social legibility turns visibility into a reputational calculation and narrows the room to seek help and to leave violent bonds. Third, mobility associated with education opens partial and reversible ruptures of this territorial enclosure. These findings extend existing scholarship in three ways. Work on metronormativity has shown that the narrative of migration to the metropolis is a normative imaginary rather than a universal trajectory [21–23], and critical geographies of sexuality have documented how non-metropolitan places generate their own forms of belonging and silencing [24–26]. The Talca–Santiago comparison adds, first, an intermediate scale that this largely Anglo-American literature rarely examines: the Latin American regional capital, which is neither rural nor metropolitan, and in which scarcity and proximity co-produce both surveillance and improvised solidarity. Second, research on rural sexual-minority health has emphasized the scarcity of services and of culturally competent care [27–29]. Our participants describe an epistemic dimension of that scarcity: when no support reference is recognizable, the question of where to seek help becomes unanswerable, and care collapses onto intimate and intracommunity circuits. Third, research on violence in LGBTIQ+ relationships has identified fear of exposure, institutional distrust, and relational isolation as barriers to help-seeking [30–33]. The comparison suggests that these barriers are territorially distributed: in narrow networks, reputation and the existence of a single available circuit make leaving a violent bond or conflict also a matter of losing one’s only community. What the comparison offers, therefore, is less a contrast between two cities than a way of showing how urban scale, network density, and the circulation of information mediate LGBTIQA+ violence and survival in a non-metropolitan Latin American setting.

The scope of the comparison is bounded by conditions that should inform its reading from the outset. The analytical sample is composed of young participants aged between 19 and 35 years, all of whom have completed or are pursuing university studies, who are predominantly cisgender and among whom there are no trans women, so that the infrastructure of possibility described here is, in part, an expression of this socio-educational position and cannot be extended to LGBTIQA+ persons in Talca and Santiago as a whole. The design is, moreover, methodologically asymmetric, since Talca, as the primary site of the broader project, contributed seven individual interviews and one focus group, whereas Santiago, conceived as a contrast site, contributed a single focus group; given that interviews and focus groups elicit different kinds of accounts, some of the differences observed between the two sites may derive from the type of data rather than from place, which is why Santiago is treated here as the contrasting horizon against which participants narrate Talca rather than as a symmetrical comparator, and why the comparison is read in analytical rather than representative terms. To these conditions are added the fact that rurality is examined primarily as biography and as circulation from urban locations, the possibility that recruitment through LGBTIQA+ organizations favored participants with greater community connection, and the differing gender composition of the focus groups conducted in each city.

## 2. Territory, subjectivation, networks, and access as territorial conditions of violence and survival

The theoretical framework articulates four interconnected dimensions for analyzing how territory operates as a condition of violence and survival for LGBTIQA+ persons. First, the approach reads territorial difference as a regime of legibility and subjectivation: city scale, network density, and information circuits set the conditions under which bodies and bonds can appear in public without immediately becoming capturable by social sanction [4,11,16,17]. Second, we draw on critical geographies of sexuality, which have argued that the urban-rural opposition operates less as a fixed cartography than as a moral-political device that distributes habitability, recognition, and risk unequally [34–37]. Within this strand, the concept of metronormativity has shown the limits of imagining LGBTIQA+ identity realization as a story of “going to the big city” [21], and recent work has insisted on the co-production of urban, suburban, and rural as morally charged categories [22–24]. Third, the framework engages with subjectivation theory [37–39] to underline that territorial conditions do not impose themselves from the outside on already constituted subjects: rather, they shape the very production of subjectivities, conducts, and survival strategies. Fourth, we mobilize work on social capital, network density, and community connection [40–43] to show that the capacity to recompose social ties after violence depends on the heterogeneity and density of available networks. This heterogeneity is unequally distributed across territories.

The article also draws on Latin American literature that resists imported diagnoses and grounds the analysis in the local production of moral and political climates around sexuality and gender [7,20,34]. The convergence of these strands enables us to read “Talca is not Santiago” not as a quantitative observation but as a practical-moral operation through which participants assess thresholds of risk, anticipate reputational costs, and design strategies of presence.

## 3. Method

### 3.1. Study design and project context

For this manuscript we analyzed a subcorpus of seven individual interviews and two focus groups. The subcorpus was drawn from a broader research project examining heterocisnormative violence in LGBTIQA+ relationships and contexts in Chile, which to date comprises 42 participants distributed across individual interviews and focus groups conducted in Talca and Santiago. We selected the subcorpus for the present analysis according to two predefined criteria: (a) density of explicit territorial references in participants’ narratives (Talca-Santiago comparisons; rurality-city contrasts; mobility trajectories), and (b) representation of both study sites. We included all cases from the broader project that met both criteria. Reporting follows the Consolidated Criteria for Reporting Qualitative Research (COREQ) guidelines (see S1 Checklist). We interpreted the corpus through Reflexive Thematic Analysis (RTA) following Braun and Clarke [44], oriented to describing patterns of meaning constructed around territorial differences (Talca-Santiago; rurality-city gradient).

*Study sites and territorial context.* Talca is the capital of the Maule Region, in south-central Chile. In the 2024 Population and Housing Census, the commune of Talca had 232,131 inhabitants and the region 1,123,008; the Metropolitan Region, which contains Santiago, had 7,400,741 inhabitants, about 40% of the national population [45]. The two regions also differ sharply in rurality. Under the commune classification of the National Rural Development Policy, based on 2017 Census data, 20 of the 30 communes of Maule are predominantly rural and 7 are mixed; together these communes cover 90% of the regional territory and house 55.7% of its population (26.8% of the regional population is rural under the census definition of the National Institute of Statistics, INE). In the Metropolitan Region, rural and mixed communes house only 4.2% of the population (3.7% under the INE definition) [46]. Table 1 summarizes these indicators together with the available evidence on discrimination.

**Table 1:** Descriptive contextual indicators for the two study sites: Talca/Maule Region and Santiago/Metropolitan Region.

Indicator	Talca / Maule Region	Santiago / Metropolitan Region	Source
Population, 2024 Census (region)	1,123,008	7,400,741	INE [45]
Population, 2024 Census (study commune)	Talca: 232,131	Not applicable (multi-commune metropolitan area)	INE [45]
Communes classified as predominantly rural / mixed / predominantly urban	20 / 7 / 3	6 / 5 / 41	ODEPA, PNDR classification, 2017 Census [46]
Population living in rural or mixed communes (PNDR), %	55.7	4.2	ODEPA [46]
Rural population, INE census definition, %	26.8	3.7	ODEPA [46]
Territory covered by rural or mixed communes, %	90	64	ODEPA [46]
SOGI discrimination cases registered by Movilh, region (2023 / 2024 / 2025)	28 / 13 / 16	224 / 209 / 179	Movilh [47]
SOGI discrimination cases registered by Movilh, commune (2023 / 2024 / 2025)	Talca: 4 / 5 / 4	Santiago: 99 / 111 / 52	Movilh [47]
Mean registered cases per 100,000 inhabitants per year, 2023–2025 (region)	1.7 (commune of Talca: 1.9)	2.8	Authors’ calculation from [45,47]
Respondents naming gay men and lesbians among the groups whose rights are least respected, ENDH 2018, %	24	29	INDH, as reported in [48]
Zamudio Law (No. 20.609) complaints, actions, or judgments on SOGI grounds, by jurisdiction	Not publicly available	Not publicly available	See text

*Note.* SOGI, sexual orientation and gender identity; INE, National Institute of Statistics; ODEPA, Office of Agrarian Studies and Policies; PNDR, National Rural Development Policy; ENDH, National Human Rights Survey; INDH, National Human Rights Institute. PNDR classifies a commune as predominantly rural when more than 50% of its population lives in census districts with fewer than 150 inhabitants/km^2^, and as mixed when this share is 25–49%. Movilh counts exclude hate speech disseminated through social media and national outlets, which cannot be assigned to a territory; they record cases known to a civil society organization and are not an incidence measure. Rates use 2024 Census populations as denominators for all three years. The indicators are descriptive and are not intended to validate the qualitative findings.

No official statistical source in Chile records violence or discrimination based on sexual orientation or gender identity (SOGI) with regional disaggregation. The most consistent longitudinal record is the annual human rights report of the Movement for Homosexual Integration and Liberation (Movilh), which compiles the cases and complaints known to the organization by region and commune [47]. Excluding hate speech disseminated through social media and national outlets, which cannot be located geographically, Movilh registered 224, 209, and 179 cases in the Metropolitan Region in 2023, 2024, and 2025, respectively, and 28, 13, and 16 cases in the Maule Region; the commune of Talca accounted for 4, 5, and 4 of these cases, and the commune of Santiago for 99, 111, and 52 [47]. Relative to the 2024 Census population, this corresponds to a mean of approximately 2.8 registered cases per 100,000 inhabitants per year in the Metropolitan Region and 1.7 in the Maule Region over 2023–2025. These figures must be read with caution. They are a registry of cases that reach a Santiago-based civil society organization, not a measure of incidence, and lower registered rates outside the capital are compatible with under-reporting where support organizations, media attention, and recognizable referral routes are scarcer. In representative survey data, the 2018 National Human Rights Survey (ENDH) of the National Human Rights Institute found that 24% of respondents in Maule and 29% in the Metropolitan Region named gay men and lesbians among the groups whose human rights are least respected in Chile [48].

Regarding Law No. 20.609 (the Zamudio Law), judicial statistics show that the anti-discrimination action it created has been used sparingly: 453 actions were filed in first-instance courts nationwide between 2012 and 2019, and, among actions filed by men between 2012 and 2016, sexual orientation was the second most frequent ground (12%) [48]. To our knowledge, however, no published judicial or administrative source reports complaints, actions, or judgments under the law disaggregated simultaneously by protected ground (sexual orientation or gender identity) and by territorial jurisdiction. We were therefore unable to compare Zamudio Law activity on SOGI grounds between the Maule and Metropolitan regions. We present these indicators not to validate the qualitative findings but to situate the institutional and empirical context in which participants narrate the Talca–Santiago difference: a smaller and substantially more rural region, in which SOGI-related discrimination is less documented, set against a metropolitan region that concentrates population, registered cases, and the organizations that record them.

### 3.2. Researcher and positionality

The principal investigator (WOAF) conducted the interviews and facilitated the focus groups. He is a clinical psychologist with doctoral training and ten years of experience in qualitative research on corporalities and LGBTIQA+ violence. He self-identifies as a homosexual man and lives in a rural area near Talca, which facilitated access to participants from the Maule Region and supported a situated understanding of the territorial dynamics analyzed. He had no prior relationship with participants. At the start of each encounter he made his position as a member of the studied community explicit and emphasized that the questions arose from academic, not therapeutic, interest.

The analytical team was completed by two co-researchers: Cecilia Del Pilar Valenzuela Gajardo and Thiare Anaís Cid Ramírez, both psychologists in training based at Universidad Autónoma de Chile (Talca campus), with experience in qualitative analysis applied to gender and sexuality research. Both co-researchers self-identify within sexual or gender minorities, are familiar with the territorial contexts under study, and have participated in community-oriented activities in the Maule Region. Beyond their analytical role, Cecilia Del Pilar Valenzuela Gajardo and Thiare Anaís Cid Ramírez also participated in the data-collection phase of the broader project. They supported fieldwork logistics (scheduling, contact with potential participants through community networks, and coordination of focus-group venues), contributed to transcription quality checks, and took part in regular team debriefing meetings in which ongoing interviews and focus-group sessions were collectively reviewed. These post-fieldwork team sessions allowed us to discuss emergent territorial references, identify potentially salient analytical threads early on, and iteratively refine the interview guide and focus-group prompts. We treated the team’s positionalities as analytical resources rather than as sources of bias to be neutralized, in line with Braun and Clarke’s reflexive epistemology [44]. Our shared situatedness in Talca grounded both data collection and analysis in lived knowledge of the region, while we systematically discussed individual interpretive emphases in team meetings to maintain analytical rigor and reflexivity.

### 3.3. Inclusion and exclusion criteria

We defined criteria a priori, consistent with the project design. Inclusion criteria: (a) identifying with non-hegemonic sexual-gender identities or LGBTIQA+ identities (not identifying exclusively as a heterosexual man or woman); (b) being over 18 years old; (c) residing in Talca or Santiago, Chile; (d) reporting having experienced situations of violence according to subjective perception; (e) having completed secondary education or, alternatively, having sufficient reading and writing skills to understand the informed consent and participate. Exclusion criteria: (a) diagnoses associated with severe alterations of reality judgment or thought flow (severe cognitive difficulties); (b) persons who exercise active violence at the time of participation.

### 3.4. Sampling strategy

We used non-probabilistic sampling, combining purposive sampling by criteria—to ensure compliance with inclusion and exclusion criteria—with snowball sampling, to expand access to eligible potential participants through community networks [49]. Because the literature has defined the LGBTIQA+ population as a hidden or hard-to-reach population [1,50], this strategy is appropriate to maximize the diversity of territorial experiences.

We decided to conclude data collection with the present corpus following the information-power framework of Malterud, Siersma and Guassora [51]. This framework holds that sample adequacy in qualitative research depends on (a) the narrowness of the study aim, (b) the specificity of the sample, (c) the use of established theory, (d) the quality of dialogue, and (e) the analysis strategy. We did not use saturation as a stopping criterion, because saturation is epistemologically inconsistent with reflexive thematic analysis [44]. In this study, the focused research aim (territorial difference in LGBTIQA+ violence and survival), the well-defined inclusion criteria, the theoretical framework drawn from critical geography and subjectivation theory, the dialogical depth between individual interviews and focus groups, and the case-oriented analytical strategy together support the adequacy of the present corpus for the research questions.

### 3.5. Recruitment

Recruitment took place between 02/07/2025 and 01/09/2025. Given the characteristics of the LGBTIQA+ population and previous studies [1,7], we recruited through articulation with LGBTIQA+ organizations in Talca and Santiago and through dissemination on social networks, with posts inviting interested persons to contact the research team directly.

We established contact with two LGBTIQA+ collectives in Talca with a territorial base in the Maule Region (one university-linked and one community-based) and one organization with metropolitan presence in Santiago. These organizations disseminated the call among their networks and, in some cases, mediated initial contact with potential participants. We recruited focus-group participants individually through these channels and through snowball referrals; they were not necessarily affiliated with the contacting organizations, and we convened them as a research team specifically for the group session. We acknowledge that recruiting through LGBTIQA+ organizations may introduce a selection effect favoring participants who are more politically engaged or community-connected; we discuss this limitation in section 5.4. To preserve the confidentiality of participants recruited through them, and to avoid identifying small local collectives whose members could become traceable in a medium-sized city, we do not disclose the specific names of the LGBTIQA+ organizations involved in recruitment. This decision is consistent with the ethical commitments approved by the institutional Ethics Committee (Protocol CEC 07–25) and reflects standard practice in qualitative research with hard-to-reach minorities in non-metropolitan territories.

### 3.6. Data collection procedure

All data collection was carried out in person and in the city of current residence of each participant: interviews and focus-group sessions with participants residing in Talca were conducted in Talca, and those with participants residing in Santiago were conducted in Santiago, in venues coordinated with the support of the LGBTIQA+ organizations described in Section 3.5. Although participants reported diverse territorial origins (including rural and intraregional trajectories, as detailed in Tables 2, Table 3–Table 4), at the time of fieldwork each participant was located in one of the two study sites.

**Table 2:** Sociodemographic characteristics of individual-interview participants.

P-E-1	18–24	University	Woman	Lesbian	Lives in Talca
P-E-2	18–24	University	Man	Gay	Lives in Talca; travels to Santiago
P-E-3	18–24	University	Transmasculine man	Heterosexual	Lives in Talca
P-E-4	18–24	University	Woman	Lesbian	Rural area of the Maule Region
P-E-5	18–24	University	Woman	Lesbian	Lives in Talca
P-E-6	18–24	University	Woman	Lesbian	Lives in Talca; travels to Santiago
P-E-7	18–24	University	Woman	Lesbian	Lives in Talca; from another commune in the Maule Region

*Note.* Ages are reported in ranges, and territorial information is generalized to protect participant confidentiality.

**Table 3:** Sociodemographic characteristics of focus-group participants, Santiago.

ID	Age	Education	Gender identity	Sexual orientation	Place of origin / Territorial trajectory
P-GF-S-1	25–35	University	Non-binary queer person	Pansexual	Santiago Metropolitan Region; intra-metropolitan contrasts
P-GF-S-2	25–35	Graduate	Man	Gay	Lives in Santiago; family of rural origin
P-GF-S-3	25–35	Graduate	Man	Gay	Lives in Santiago; rural upbringing
P-GF-S-4	18–24	University	Transmasculine non-binary person	Bisexual	Lives in Santiago
P-GF-S-5	25–35	Graduate	Gender-fluid non-binary person	Pansexual	Lives in Santiago; family with rural customs

*Note.* Ages are reported in ranges, and territorial information is generalized to protect participant confidentiality.

**Table 4:** Sociodemographic characteristics of focus-group participants, Talca.

ID	Age	Education	Gender identity	Sexual orientation	Place of origin / Territorial trajectory
P-GF-T-1	18–24	University	Man	Gay	Lives in Talca; rural origin in the Maule Region
P-GF-T-2	18–24	University	Woman	Lesbian	Born in Talca; lived in other regions before returning
P-GF-T-3	18–24	University	Woman	Lesbian	Lives in Talca; from another commune in the Maule Region
P-GF-T-4	18–24	University	Woman	Lesbian	Lives in Talca
P-GF-T-5	18–24	University	Woman	Bisexual	Lives in Talca; from another commune in the Maule Region
P-GF-T-6	18–24	University	Woman	Bisexual	Lives in Talca
P-GF-T-7	25–35	Graduate	Non-binary person	Pansexual	Lives in Talca

*Note.* Ages are reported in ranges, and territorial information is generalized to protect participant confidentiality.

*Individual interviews.* We conducted seven semi-structured interviews in person. Participation included between one and three sessions per participant, with a total duration per participant of between 45 and 180 minutes (depending on the number of sessions). All interviews were recorded with authorization and subsequently transcribed for analysis.

*Focus groups.* We conducted two focus groups in person, one in Talca (7 participants) and another in Santiago (5 participants). Each focus group lasted approximately 2 hours and 30 minutes. We recorded the sessions with authorization and subsequently transcribed them.

*Team-based fieldwork and post-session debriefings.* Although the principal investigator facilitated the individual interviews and the focus groups, we conducted the broader data-collection process as a team effort. Cecilia Del Pilar Valenzuela Gajardo and Thiare Anaís Cid Ramírez participated in fieldwork support tasks (coordination of contact with potential participants through community networks, scheduling, and logistics for the focus-group venues) and in transcription quality checks. After each interview and each focus-group session, the three team members met for debriefings in which we jointly reviewed the recordings and field notes. These sessions served three purposes: (i) to identify recurrent territorial references and tensions worth exploring further; (ii) to refine the interview guide and focus-group prompts for subsequent sessions; and (iii) to reflect on the interactional dynamics observed during each encounter, including the affective tone of participants’ narratives. These team-based debriefings shaped both the conduct of subsequent fieldwork and the analytical work described in Section 3.7.

The methodological asymmetry between the two sites (seven individual interviews and one focus group in Talca; one focus group in Santiago) reflects the design of the broader research project. Talca operates as the primary site, given the centrality of medium-sized cities to the project’s territorial argument and the team’s situated access to local networks. Santiago functions as a contrast site that anchors the comparative analysis. We acknowledge that individual interviews and focus groups generate qualitatively different kinds of data: individual interviews allow for greater privacy, depth, and personal narrative development, which is particularly relevant for sensitive topics such as violence, whereas focus groups foreground intersubjective construction and collective meaning-making. The comparative analysis between cities therefore rests on asymmetric methodological footing, a limitation we discuss explicitly in section 5.4.

### 3.7. Data analysis

We followed the six phases of Reflexive Thematic Analysis [44]: (1) familiarization with the data through repeated reading of transcriptions; (2) generation of initial codes oriented to identifying territorial references, experiences of surveillance and visibility, and mentions of networks, spaces, and information; (3) search for themes by grouping codes into patterns of meaning; (4) review of themes verifying internal coherence and distinction between themes; (5) definition and naming of themes; (6) report production. The principal investigator and two co-researchers performed coding as a team, which allowed us to deepen the analysis and to triangulate interpretations. We conducted analyses through iterative review sessions of the material to ensure that the themes adequately represented the corpus.

To make the triangulation process explicit: after independent familiarization with the full corpus, the three team members each generated initial codes on a shared subset of transcripts. We then discussed these codes in consensus-building meetings, refined definitions, merged redundancies, and developed a working codebook. We applied the codebook collaboratively to the remainder of the corpus, holding weekly meetings during the analytic phase to discuss code applications, emerging patterns, and candidate themes. We resolved disagreements through dialogue, returning to the transcripts and to the theoretical framework. Consistent with Braun and Clarke’s reflexive epistemology [44], we did not use inter-coder agreement metrics. We treated divergence among coders as productive of interpretive depth, and team triangulation served to surface and discuss multiple readings rather than to enforce convergence. The full codebook is publicly available (S2 File; see Data Availability Statement).

A further consideration concerns the inductive grounding of the analysis. Although the study mobilizes a substantive theoretical framework drawn from critical geography and subjectivation theory, we did not construct the themes top-down from this framework. During early coding, the team generated a substantially broader set of initial codes than those that ultimately found their way into the final themes. We generated codes addressing dimensions such as economic violence, cultural and political dynamics, and access to specific institutional spaces (the university and paid work in particular), but we progressively retired them because their density in the corpus was insufficient to sustain robust patterns of meaning, or because variations across cases were too heterogeneous to consolidate under a single theme. Retirement was not a matter of theoretical convenience but a consequence of returning iteratively to the transcripts and asking whether the data sustained the proposed pattern. In some cases, content originally coded under these categories was reabsorbed into the three themes finally reported (for example, references to economic dependency that intersect with the discussion of leaving violent bonds in Theme 2, or references to the university as a space of partial opening within Theme 3). This pruning is consistent with the reflexive epistemology of RTA [44], which treats theme construction as a situated interpretive act anchored in the corpus rather than as a derivation from theory. We make this analytic history explicit to address the legitimate concern that theoretical commitments could have overdetermined the analysis: the three themes finally reported are those for which the corpus, read across coders and through iterative team discussion, sustained dense and analytically articulated patterns of meaning.

### 3.8. Ethical considerations

Participation was voluntary, and we obtained written informed consent (handwritten signature) prior to data collection. We informed participants about the study objective, procedures, voluntariness, confidentiality, possible risks and benefits, and the right to withdraw at any time without consequences.

*Ethical approval.* The study has approval from the Ethics Committee of Universidad Autónoma de Chile (Protocol No. CEC 07–25, approved on 20-03-2025).

*Confidentiality and anonymization.* We anonymized transcriptions and quotations: we eliminated or substituted proper names and potentially identifying data, and used alphanumeric codes for quotations in the manuscript (e.g., P-E-1, P-GF-T-2, P-GF-S-3). We stored audio files and transcriptions on an encrypted personal computer, with access restricted to the research team.

## 4. Results

The reflexive thematic analysis organized the corpus into three themes that synthesize how participants narrate the Talca–Santiago difference and the rurality–city gradient when speaking about access to information, availability of networks and spaces, experiences of social surveillance and reputation, and effects of mobility associated with studies and trajectories. Across the themes, we integrate interviews (E) and two focus groups: FG Talca (7 participants) and FG Santiago (5 participants). In each theme we present quotations with anonymized coding. At first appearance, each individual-interview participant is identified by code, gender identity, sexual orientation, and city, so that the territorial-identity coordinates of each quotation are available to the reader without requiring repeated reference to Tables 2, Table 3–Table 4.

### 4.1. Theme 1. Infrastructure of possibility: information, networks, and spaces

This theme gathers accounts in which territorial difference operates as variation in three concrete registers: what information arrives, which support references exist, and which spaces or activities are available for socializing, finding peers, and sustaining a continuity of daily life. The analytic argument is that these three registers do not work as separate items but as a single infrastructure that makes LGBTIQA+ life practicable—or, in its absence, precarious—at a given urban scale.

*Information and language available to name the experience.* The first register concerns whether the cultural-linguistic resources to name one’s own experience arrive at all. In the Talca FG, one participant links his early socialization to a Catholic and semi-rural context and describes that for years he did not know “how to put it into words” or “have the tools” to name what he felt: “because I was at this Catholic school, because it’s a more rural context, the topic wasn’t discussed, so I didn’t know; I only found out around 12–13 years old what it was, what it meant to be gay. I never had the language, the tools to say what I was feeling” [P-GF-T-1]. The lack of language is not merely an absence; it is a positive effect of a territorial-religious configuration that actively forecloses certain vocabularies, with measurable lag effects on self-understanding. The same regional configuration is indexed by other Talca-FG participants who anchor themselves through territorial trajectory: “I’m from a town near Curicó […] and I recently moved to Talca” [P-GF-T-1]; “I’m from Constitución and I’ve been living here in Talca for about 6 years” [P-GF-T-3]; “I’m from Chanco […] a rural little town, countryside, countryside” [P-GF-T-5]. These introductions are analytically significant because they locate the speakers along the intraregional gradient from rurality to medium-sized city, the gradient along which information and recognition circulate unevenly.

In the Santiago FG, participants describe access to information as similarly unequal, even within the metropolitan region. P-GF-S-1 formulates the difficulty in terms of “certain trends arriving” to places with “so little communication,” noting that “the only thing I had to know those things was radio. I will never hear to this day on AM Radio anything related to LGBT, for example.” The same participant complicates the metropolis-rurality opposition itself: “living in Providencia is very different from living in Maipú or Lampa, and the metropolitan region also has ‘super rural areas’” [P-GF-S-1]. The complication is analytically important: the infrastructure of possibility does not align cleanly with the administrative boundary of the city; it is internally stratified by *comuna* and by the density of media circuits that actually reach each location.

*The existence of recognizable support references.* The second register concerns the presence or absence of “references” to support networks that can be named. One participant marks the Talca-Santiago difference precisely as a difference of “having a reference” and “knowing” the network: “Here I don’t know any support network… in Santiago I know Movilh… like I have that reference that it exists in Santiago, but here in Talca I don’t” [P-E-6, lesbian woman, Talca]. Another interview converges on this absence: P-E-3 reports being unfamiliar with “help centers” and locating support primarily in friendship—“Like… help centers or I don’t know any. […] like generally I talk about it a lot with my friends” [P-E-3, heterosexual transmasculine man, Talca]. What is at stake here is not only a quantitative scarcity of services but an epistemic limit: in the absence of recognizable references, the question “where do I go?” becomes unanswerable, and care collapses back onto the intimate circle. P-E-2 narrates a parallel response to this absence in the form of a self-organized infrastructure: “here in Talca there are a couple of groups I’m part of. They’re self-organized groups. A person from Talca had the idea of making a group of people who belong to the community to provide support among themselves” [P-E-2, gay man, Talca]. The contrast that organizes the theme is therefore not between “support” and “no support” but between *referred* and *improvised* support: in the medium-sized city, support exists, but it must be invented in the absence of institutional references.

*The availability of spaces and the visibility of biographical horizons.* The third register concerns the availability of physical spaces and scenes where LGBTIQA+ life is visible across ages, occupations, and forms of partnership. P-E-6 narrates her first visit to a lesbian bar in Santiago as a moment in which the *imaginability* of an LGBTIQA+ future expanded: “I went for the first time to a lesbian bar and I said… this has to be in Talca… I saw old lesbians for the first time… lesbians in work uniforms… it’s no longer a youth thing… there can be lesbians at different stages of life… it’s like cool to know that there’s something after youth” [P-E-6]. The analytic point is that what the metropolitan scene supplies is not entertainment but *biographical evidence*: it makes a long-term lesbian life visible as something other than a youthful interlude. In the same direction, P-E-2 contrasts the quantity of circuits between the two cities: “In Santiago there are many more activities… here in Talca we are much more limited” [P-E-2], and explains the practical consequence in terms of the freedom to leave a group when a group becomes intolerable: “In Santiago… you have ten more *comunas* to move around… you can hang out with another group of people… but here… ‘here’s the group and you have to settle for this group’” [P-E-2]. The scarcity of available circuits, in this reading, is not merely an aesthetic constraint; it locks participants into the only available group, which is precisely the condition under which intracommunity conflict cannot be exited.

*Family restrictions as filters on the infrastructure.* The theme also encompasses how family fears and sanctions filter access to the infrastructure that exists. P-E-5 describes that her participation in cultural spaces was forbidden out of fear of physical violence: “afterward I couldn’t go because my parents didn’t let me, for fear that […] if my dad found out, even that they’d hit me” [P-E-5, lesbian woman, Talca]. The same participant locates the religious-school environment as a parallel filter: “it was complicated because it was a religious school […] they almost expelled me for that” [P-E-5]. In the Santiago FG, P-GF-S-2 articulates the inverse direction of this same filter, namely how educational access opened the family climate over time: “the first thing that comes to mind is access to education… in my generation several of us entered university, or got to know institutes… we started to know other contexts” [P-GF-S-2, gay man, Santiago]. Educational mobility, in his account, did not only change the individual’s horizon; it shifted the family configuration itself, which then ceased to operate purely as a filter.

*Nuancing the linear rurality-restriction association.* The theme also contains accounts that complicate any linear association between rurality and closure. P-E-4 narrates that her extended family in Purísima, including grandparents and aunts over 60 years old, did not exercise violence or negative comments about her homosexuality: “My grandfather […] cried because he was afraid something would happen to me. […] He says he still sees on TV when something happens. […] And I tell him, and you, what if something happens to you? And I’d die. And he tells me, yes, you are my grandchildren. I love you however you are” [P-E-4, lesbian woman, rural Maule (Purísima)]. This case is not anecdotal: it shows that the rural context does not operate as an absolute determinant. The infrastructure of possibility is articulated with specific family configurations whose internal histories—including specific relationships of trust between generations—do not always align with the territorial regime. Conversely, the conservative-professional environment of a smaller-niche labor market can re-impose restriction even on participants located in the medium-sized city: “I identify as a non-binary person but more feminine-leaning. Why? Because of my job, you’ll know that the midwifery field is quite conservative. So I can no longer have a gender expression that I’d like” [P-GF-T-7]. Here the constraint is not the city as a whole but a sector within it—a reminder that the infrastructure of possibility is also stratified by professional field.

### 4.2. Theme 2. Social surveillance and reputation: feeling observed, rumor, and daily control

This theme gathers accounts where participants describe daily visibility as exposure to gazes, comments, “things they make up,” and social control linked to environments where people know each other. The analytical claim is that, in medium-sized cities and adjacent ruralities, social legibility intensifies daily surveillance and reorganizes visibility as reputational hyperexposure: visibility becomes not an option but a calculation. The theme is structured around four operations of moral regulation: ambient observation; rumor as reputational damage; intraregional moral gradients; and intracommunity weaponization of identity.

*Ambient observation and scale.* P-E-6 articulates surveillance as a sensation of being watched, anchored in the very scale of Talca: “In Talca… ‘not holding hands because I feel very watched’… ‘here everyone knows everyone’… Talca, a smaller place, becomes more dangerous” [P-E-6]. The analytic move at work in her account is to link three propositions: city scale → universal acquaintance → enhanced danger. Smallness is not described in aesthetic terms but as a property that converts public space into a continuous field of recognition, in which the body becomes legible to anyone, anywhere, at any time. In the Santiago FG, P-GF-S-1 names this same condition through the Chilean idiom and links it to material infrastructure: “It’s like this thing about small town, big hell… there were places where… they didn’t arrive… not even to call by phone… I was very scared… ‘if something happens to me, how do I… ask for help?’” [P-GF-S-1]. The fear here is not abstract; it is anchored in the absence of phone signal, which makes asking for help operationally impossible. Surveillance and the absence of help-circuits, in this account, are two faces of the same territorial configuration.

*Rumor as reputational damage and avoidance of community spaces.* P-E-3 describes the effect of rumor as a force that pushes him out of the local LGBT community itself: “they’ve made up things about me, so like I don’t feel so comfortable within the LGBT community here in Talca” [P-E-3]. The interpretive point is critical: rumor does not only sanction transgression of heteronorms from outside the community; it also reorganizes the internal life of the community, producing avoidance of the very spaces that should function as support. P-E-4 records the more diffuse but persistent register of the same operation—“Just looks” and “They’ve said things to me” [P-E-4]—signaling that surveillance in the medium-sized city does not require dramatic events to do its work. It is its very ordinariness that makes it a sustained discipline.

*Moral regulation as an intraregional gradient.* The theme also surfaces a fine-grained territorial differentiation that runs *within* the Maule Region. P-E-7 contrasts Talca and Linares and formulates this contrast as a difference in what counts as legible affection: “Here in Talca… my professors know… they see it as a bit more normal… But in Linares, it remains a taboo topic… ‘Displays of affection, no’” [P-E-7, lesbian woman, Talca (origin Linares)]. The same participant locates this difference in a specific generational composition: “in Linares there’s a lot of older adult population. […] You can notice it in the actions they have with you. For example, it happened to me that I recently went with my girlfriend to Linares […] and the other one being feminine. But here in Talca it’s like, well, it also depends” [P-E-7]. The analytic significance of these accounts is that the Talca-Santiago axis does not exhaust the territorial argument: a comparable moral-geographic operation organizes the intraregional axis Talca-Linares. The same logic of legibility, generational density, and moral threshold reappears at a finer scale, which is one of the reasons why the article resists collapsing the territorial argument into a simple urban-rural binary.

*Family environments and hegemonic masculinity as corrective infrastructure.* Inside the home, the same surveillance operates through corrective directives that target gestures, voice, and conduct. P-E-1 describes a sustained pressure to perform hegemonic masculinity: “It was always like, no, don’t do this. Don’t move like that, don’t talk like that, don’t do this. […] Even doing household chores. Like he’d say things to me” [P-E-1, lesbian woman, Talca]. The list-form of her account is itself analytically significant: surveillance disciplines a fine grain of conduct, not only sexual identity in the abstract but the micro-mechanics of body and voice. Institutional violence reproduces the same logic in the professional sphere; P-E-7 describes an internship in which the supervisor announced an explicit corrective project: “no, it’s just that you’re not normal. […] Openly. And it was like, I don’t like people like that, so I’m going to do everything possible to get you kicked out of the internship” [P-E-7]. The continuity between domestic and institutional registers indicates that the corrective logic is not site-specific; it is a portable repertoire that moves with the subject across her territorial circuits.

*Intracommunity weaponization of identity.* Surveillance does not stop at the boundary of the LGBTIQA+ community; it crosses into it and acquires specific weapons. P-GF-S-4 describes how deadnaming was deployed within an explicitly LGBTIQA+ academic environment, by peers who shared identity coordinates with him, as a retaliation: “they started to misgender me. And I was like, what? And most of the people in my career, if not 95% more or less, are all gay people, community people, and that for me was super violent because I, this is my fifth year as student council, I’ve been in the career for five years and this is my fifth year as student council and gender delegate on top of that, so no, you lost, you can’t come and misgender me” [P-GF-S-4]. The same operation appears in the family register, in the form of deadname-as-weapon: “when they get angry, I mean, this applies to my younger siblings, my older siblings and my parents, they get angry and coincidentally they call me by my deadname. And that’s like damage times 10, I don’t know. So I honestly think they do it deliberately, like going directly at a person’s identity” [P-GF-S-5]. The analytic claim that the theme sustains is that intracommunity violence is not the *opposite* of surveillance but its internal extension: the very identity coordinates that the community is supposed to recognize become the precise points at which violence is administered.

### 4.3. Theme 3. Mobility and education as partial opening: Displacements, trajectories, and recomposition

This theme organizes accounts in which movement between territories—rurality, medium-sized city, metropolis—particularly when associated with studies, operates as an opening of options, access to encounters, and reconfiguration of available circuits. The analytical claim is that mobility and education function as a *partial* rupture of the territorial enclosure: they expand horizons but do not erase the access asymmetries that condition the opening in the first place. The theme is structured by three articulations: educational mobility as biographical opening; the metropolis as access to specialized circuits; and the limits of the opening.

*Educational mobility as biographical opening.* In the Talca FG, P-GF-T-1 formulates the link between studies, family migration, and biographical opening in compact form: “We started coming little by little as people started studying… ‘Like studying made social mobility’” [P-GF-T-1]. The analytic move here is that educational mobility is not narrated as an individual project but as an intergenerational rearrangement of the family’s relationship to territory. The same logic appears in the Santiago FG, where P-GF-S-2 narrates the trajectory from a poor, rural-rooted family to professional life and identifies education as the principal mechanism that opened other contexts: “In my family, we’re a lot, I mean, my uncles are twelve, of those twelve several have five or six children, there are a lot of us because we’re a poor family and on top of that half from the countryside, so there’s a very, very, very lacking context in many things at one point. But in my generation… there are several who entered university, or I don’t know, we got to know institutes, but like we started to know other contexts” [P-GF-S-2]. Education, in his account, did not erase the family’s class position but produced a generational shift in the available repertoires of meaning and contact.

*The metropolis as access to specialized circuits.* Educational mobility frequently runs in parallel with episodic travel to the metropolis to access events and scenes that are simply unavailable in the medium-sized city. P-E-2 describes a transmasculine encounter in Santiago in terms that combine specificity and affective intensity: “I went… to Santiago… to a transmasculine encounter… it was a very beautiful experience… ‘an almost utopian thing’” [P-E-2]. The extended account makes explicit what was specific about the encounter: “they were all people belonging to the community, transmasculinities, binary and non-binary, and the experience, at least, for what I lived, what I could see, was an almost utopian thing, like for me it wasn’t real” [P-E-2]. The word “utopian” is analytically significant here: it indexes the gap between what is *imaginable* in the medium-sized city and what is *experienced* in the metropolitan event. The metropolis, in this register, does not function as permanent residence but as access to a specialized circuit that retroactively reveals the limits of the territory of residence. P-GF-S-3 articulates a related but distinct trajectory: arriving in Santiago at 18 from a rural origin allowed him to encounter people who “don’t fit into your square” precisely because the urban density supplies friction with difference: “in the countryside people are more isolated, so like generally a more conservative thinking, more right-wing, so God, country and family, the whole thing. But in Santiago, like I feel there’s more friction, like basically even if families are more conservative, you still see people on the metro, on the street, at work, anywhere else, basically there are people who don’t fit into your square” [P-GF-S-3]. The metropolis, in his reading, does not have to *want* to be open; its sheer density of strangers produces an exposure to difference that the rural locality cannot supply.

*The limits of the opening.* The same participant who narrates the utopian encounter also names its limit: returning to Talca, the available group is again a single group. “In Santiago… you have ten more *comunas* to move around… you can hang out with another group of people… but here… ‘here’s the group and you have to settle for this group’” [P-E-2]. The opening, in this account, is real but reversible: it does not migrate back with the participant. P-E-1 describes the university as the local equivalent of this opening—“Already at university I could live my life and can be who I am without any fear” [P-E-1]—but the same participant locates the previous four years of Catholic boys’ high school as the prior constraint that the university partially undid: “they were like four very difficult years, very difficult” [P-E-1]. Mobility produces openings, but these openings are conditioned by what came before; they are partial precisely in that sense.

*Local recomposition through digital coordination.* The theme also gathers accounts that resist any reading of Talca as pure scarcity. P-E-2 describes the WhatsApp group as a concrete infrastructure of recomposition: “I feel that… in smaller cities people support each other more… there’s a WhatsApp… of people from Talca… where they have gatherings, they have meetings…” [P-E-2]. The analytic point is that the same scarcity that locks participants into the only available group also generates *situated solidarities*: the proximity that makes the medium-sized city a site of surveillance is also what makes peer support possible. The theme therefore sustains a productive tension that the discussion will pick up: the medium-sized city is not only restriction; under specific conditions, it is also a site where alternative infrastructures are improvised precisely because formal ones are scarce.

## 5. Discussion

The findings suggest that participants narrate violence differently in relation to Talca and Santiago, and along the rurality-city gradient, and that they situate territory as an active condition of possibility, not as a backdrop. In dialogue with critical geographies of sexuality, the findings show that urban scale operates as a situated regime of legibility and access: it organizes where appearing becomes tolerable or punishable, and it differentially distributes anonymity, network density, information circulation, and availability of community scenes [52]. In this sense, the statement “Talca is not Santiago” is not a quantitative observation but condenses a territorial grammar in which LGBTIQA+ life becomes practicable under unequal conditions, in tension with metronormative narratives that associate identity realization with “going to the big city” [21]. At the same time, the accounts in the corpus complicate the urban-rural opposition by surfacing gradients and internal reconfigurations (for example, intraregional differences and mobility trajectories), in line with evidence that urban, suburban, and rural are co-produced as morally charged and politically contested categories [23,24]. The Talca-Santiago comparison therefore functions less as a fixed map than as a practical-moral operation through which participants assess risk thresholds, sustain strategies of presence, and anticipate reputational costs. This reading converges with work showing that the non-metropolitan produces specific forms of agency, belonging, and silencing, rather than mere absences [25,26].

### 5.1. Infrastructure of possibility: Information, networks, and spaces

The first theme shifts the discussion from visibility as an individual decision to visibility as a territorially produced condition. Access to language to name the experience, the arrival (or not) of information through media and connectivity, “having a reference” of support networks, and the availability of activities and spaces all appear as practical thresholds for imagining help, biographical continuity, and belonging. This reading converges with research that documents health and well-being disparities of sexual minorities in rural areas, associated with the unequal distribution of resources, information circuits, and culturally competent services [27,28], and with recent evidence that stigma and local climate are linked to differences in mental health and use of care in sexually and gender diverse populations in rural contexts [29]. In parallel, the manuscript shows that the lack of “references” for support is not experienced as abstract data but as an epistemic and relational restriction that narrows what is even thinkable as a course of action. By contrast, the greater density of scenes in the metropolis expands repertoires without guaranteeing homogeneous inclusion, because urban infrastructure is itself stratified by inequality and belonging.

The findings on situated support practices—self-organized groups and coordination through messaging applications—converge with literature that identifies community connection as a protective factor [40], although not without tensions. Digital technologies appear as ambivalent infrastructure: they can compensate for territorial isolation and facilitate bonding, but they also amplify traceability and risks of exposure in intense surveillance environments [53,54]. Everyday micro-spaces, including the home, work as protective and as scrutinized at the same time, in line with analyses that conceptualize the queer home as an ambivalent space of security and control [55]. Read together, by treating infrastructure as a material-discursive assemblage rather than as “equipment,” the article aligns with recent calls to conceive infrastructures as urban and territorial conditions of access, care, and possibility, particularly for minorities [56]. The findings also dialogue with evidence on sexual-minority adolescents in rural contexts, where access to support and recognition is conditioned by place and by the availability of community resources [57,58], and with contributions oriented to designing LGBTIQA+ rural futures considering local needs and participation circuits [59].

### 5.2. Social surveillance and reputation

The second theme suggests that, as participants narrate the medium-sized city and its adjacent ruralities, social legibility intensifies daily surveillance and reorganizes visibility as reputational hyperexposure. Gazes, comments, rumor, and taboo work as moral technologies of correction that shrink the margin to maneuver, including the conditions for asking for help without activating secondary sanctions. This dynamic is intelligible from theories of stigma that underline its relational and structural character: the mark does not reside in the individual but in social configurations that produce difference as devaluation [60]. At the territorial level, stigmatization is anchored in neighborhood and locality reputations, which reinforces inequality and avoidance—a process conceptualized as spatial stigma and place-based stigma [61,62]. The relationship between urban size and anonymity helps explain why “small town” is experienced as a reduction of options: relational density amplifies traceability and raises the cost of managing information about intimate life [63]. In psychosocial terms, the anticipation of sanction and identity management in hostile contexts is linked to evidence on internalized stigma and self-management strategies that affect well-being and help-seeking [64,65]. These processes connect with the concept of structural stigma, where policies, institutional climates, and local norms configure conditions that produce health and safety inequality for sexual minorities [66–68].

A specific contribution of the manuscript is to show that reputation does not operate only as “opinion”: it operates as moral infrastructure that administers circulation and contact. Rumor functions as a mechanism of discipline and belonging, consistent with approaches that conceptualize gossip as a social technology that coordinates norms, punishes transgressions, and regulates cooperation [69–72], and with work that links it to processes of boundary-making and belonging [73]. Within this framework, avoidance of local community spaces for fear of “things they make up” is intelligible less as a lack of desire for community and more as a rational calculation in the face of exposure costs in narrow networks. In parallel, the search for formal help becomes territorialized: the decision to report or activate institutions is mediated by sexual identity and risk assessment, in line with evidence on LGBTI variations in crime reporting and institutional contact [74].

These readings require a caveat that bears directly on the city-to-city comparison. Most accounts of reputational surveillance come from the Talca individual interviews, a format that affords the privacy and narrative depth in which such experiences are more readily disclosed. The Santiago material comes from a single focus group, in which participants may be less inclined to narrate intimate episodes of rumor or exposure before peers. The greater salience of surveillance in the Talca material may therefore partly reflect this difference in data type rather than a difference between the two cities. We accordingly read the contrast as a difference in how participants narrate these territories, not as a measured difference in the prevalence or intensity of surveillance.

### 5.3. Mobility and education as partial opening

The third theme describes mobility and education as a partial rupture of the territorial enclosure: moving for studies or traveling to the metropolis enables encounters, expands repertoires, and reconfigures future horizons, but does not eliminate access asymmetries. This is consistent with literature showing that the mobility decisions of gay and lesbian persons are crossed by family ties and resources, so that migration does not function as a universal “exit” but as a differentially available strategy [75]. Evidence on migration intentions among rural adolescents suggests that sexual orientation can interact with perceptions of opportunity, belonging, and local well-being, reinforcing the anticipatory dimension that the manuscript captures when participants describe Santiago as a horizon of possibility and Talca as a partial enclosure [76]. In Global South contexts, scholars have warned that sexual and gender mobilities are inscribed in territorial inequalities and differential opportunities, against linear readings of “center/freedom” versus “periphery/restriction” [77]. Rurality is also associated with specific normative climates in public opinion on LGBTIQ+ rights, which helps explain why “appearing” is calculated differently across territories [78]. The key finding, however, is that mobility produces situated and reversible openings: it enables access to events and scenes, but return or permanence in medium-sized territories requires recomposing social life under conditions of high legibility. At that point, the density and heterogeneity of networks, as forms of social capital, are central to understanding why, in contexts of scarcity, depending on a single circuit can intensify vulnerability, while having alternatives facilitates recomposition [41]. This reading converges with studies on belonging in towns and non-metropolitan cities that show how the politics of “community” can produce support and exclusion at once, depending on how sexual difference is negotiated [79–82]. The manuscript also sustains a productive tension: Talca does not emerge only as restriction, but also as a place where, by proximity and scarcity, situated solidarities are built; this double face becomes intelligible when we consider that community connection can buffer minority stress, although it is exposed to surveillance and rumor dynamics [40,42,43].

A relevant nuance must be made explicit at this point: the infrastructure of possibility described throughout this corpus is not a purely territorial effect. It is co-produced by class and educational position. The capacity to travel to Santiago for community encounters, to coordinate through WhatsApp groups, to name and recognize LGBTIQA+ experiences, or to imagine mobility as a horizon depends materially on resources, time, and educational trajectories that are unequally distributed. Participants themselves problematize this articulation. P-GF-S-2 names access to education as the principal tool that made other contexts available: “the first thing that comes to mind is access to education… in my generation several of us entered university, or got to know institutes… we started to know other contexts… the main tool I had… is the access to know new things” [P-GF-S-2, gay man, Santiago]. P-GF-S-1 articulates the converse: arriving at university from conditions of material disadvantage reorganizes recognition *within* LGBTIQA+ spaces themselves: “for someone LGBT and queer-non-binary to be arriving at university coming from a poor family, from a rural area or from the western side of the city, public schools […] visibility is given mostly to queer people who also have resources […] the socioeconomic, educational, even sociodemographic level generates greater visibility” [P-GF-S-1, non-binary queer person, Santiago]. The same participant adds that being read as “the young queer” within activist circles was repeatedly accompanied by being looked down on and having one’s work invalidated. Class and generational position, in this account, stratify recognition even within communities that are nominally aligned in their struggle.

These accounts indicate that what this article has called “infrastructure of possibility” should not be read as a pure function of urban scale. Class operates as a primary protective condition: greater economic resources are associated with softer thresholds of exposure and risk, broader access to support circuits, and wider margins of recomposition after violence. Education functions as a mediating mechanism that activates this protection by opening informational circuits, vocabularies, and networks with which to name and resist violence. But education also operates as a marker that internally stratifies LGBTIQA+ communities, producing internal hierarchies of recognition. In this expanded reading, the statement “Talca is not Santiago” names not only a moral geography of territorial scale but a moral geography intersected by class and educational position, in which what becomes practicable for some remains impracticable for others within the same territory. This is one of the reasons why the findings of this study must be understood as referring most directly to a specific socio-educational stratum—young, university-educated participants—rather than to LGBTIQA+ persons in Talca and Santiago as a whole, a calibration we elaborate in Section 5.4.

Although the empirical focus of this subcorpus emphasizes territory, information, surveillance, and mobility, the findings have direct implications for understanding violence and the possibility of “leaving” damaging bonds or circuits. The available literature shows that violence in LGBTIQ+ relationships presents relevant patterns and specific barriers to help, including fear of exposure, institutional distrust, and relational isolation [30,31]. In particular, studies on violence in sexual-minority women’s couples show that minority stress and substance use can intertwine with vulnerability to intimate partner violence, while help-seeking is hindered by stigma and inadequate services [32,33]. The specific contribution of this manuscript is to show that these barriers are not only psychosocial; they are territorialized. In medium-sized cities and adjacent ruralities, reputation and traceability restrict the possibility of asking for help without being publicly exposed; the scarcity of circuits increases dependence and can hinder recomposition after leaving a bond. In parallel, the finding about insufficient connectivity and fear of not being able to request assistance suggests that material infrastructure (phone network, transportation, routes) is integrated with moral infrastructure (rumor, surveillance) to produce differentiated survival conditions.

In applied terms, the results show that intervening in LGBTIQA+ violence and survival requires strengthening information and support infrastructures in medium-sized cities and adjacent ruralities: clear referral routes, culturally competent services, accompaniment devices that minimize reputational exposure, and sustainable community spaces that do not depend exclusively on consumption or mobility. The ambivalent character of digital technologies also suggests that care strategies must consider both their potential for connection and their risks of traceability in high-surveillance environments [53,54]. The article opens a comparative agenda oriented to evaluate territorial configurations within regions (medium-sized city versus smaller cities and rurality), to examine variations by life-course stage, and to specify the mechanisms by which rumor, reputation, and network density affect the possibility of leaving violent bonds and recomposing social life without becoming capturable by local sanctions.

### 5.4. Limitations

Several limitations of this study deserve explicit consideration.

First, the composition of the analytical subcorpus sets important boundaries for the scope of the findings. Participants are predominantly young (19–35 years), and all have completed or are currently enrolled in university education. There is representation of non-binary and transmasculine identities, but the sample does not include trans women, who are arguably the population for whom the dynamics of bodily legibility and territorial sanction operate most acutely—particularly in medium-sized cities and rural contexts where the management of visibility is more difficult. The infrastructure of possibility described in this study—circuits of information, mobility through education, WhatsApp coordination, and access to community scenes—is, in part, a feature of being young, university-educated, and predominantly cisgender, not only of being LGBTIQA+ in these territories. The findings should therefore be understood as speaking to this specific demographic profile and not to LGBTIQA+ persons in Talca and Santiago more broadly. A trans woman in her fifties, or an LGBTIQA+ person without higher education residing in a small town near Talca, likely inhabit territorial realities that this study cannot speak to. Future research must prioritize trans women, older adults, and LGBTIQA+ persons without university education in medium-sized cities and ruralities.

Second, there is a methodological asymmetry between the two sites: Talca contributed seven individual interviews and one focus group, while Santiago contributed only one focus group. Although this asymmetry is justified by the design of the broader research project—in which Talca operates as the primary site and Santiago as a contrast site—it affects the comparative depth of data available for each location. Individual interviews and focus groups generate qualitatively different kinds of data: individual interviews allow for greater privacy, depth, and personal narrative development, especially regarding sensitive topics such as violence, whereas focus groups foreground intersubjective construction. The comparative analysis between cities therefore rests on asymmetric methodological footing, and certain dimensions of the Santiago experience may be underrepresented in relative depth.

Third, the operationalization of rurality in this study warrants clarification. Although the theoretical framework engages extensively with the rurality-city gradient, the comparison is conducted between two cities (Talca and Santiago). In the corpus, rurality appears primarily in two registers: as biographical narrative (many participants come from or were raised in rural or semi-rural areas but currently reside in urban centers) and as a gradient of present circulation (some participants continue to move actively between rural localities and Talca). The findings therefore address how rurality is invoked, remembered, and lived from urban locations, rather than rurality as an exclusive current condition of residence. Studies that include participants currently residing in rural localities are necessary to complement this analytical scope.

Fourth, recruitment through LGBTIQA+ organizations may have introduced a selection effect favoring participants who are more politically engaged or community-connected. This may have shaped the visibility of certain themes (such as activism, self-organized groups, and identity articulation through community-based language) and may have reduced the representation of LGBTIQA+ persons who do not articulate their experience through community-based organizations.

Fifth, the manuscript is prepared for international publication in English, while all data were collected and analyzed in Spanish. Although we performed translations with care for nuance and idiomatic specificity, certain culturally embedded expressions (regional terms, Chilean idioms, affective registers) may have lost subtle layers of meaning. The original Spanish quotations are available from the corresponding author upon reasonable request.

Sixth, this study uses a qualitative interpretive design and does not aim at statistical generalization. The constructed themes are situated representations of participants’ meaning-making in specific territorial contexts; their transferability to other contexts requires careful consideration of comparative conditions.

Seventh, the gender composition of the data-collection encounters is itself a methodological consideration worth making explicit. Across the corpus, women predominated in the individual interviews (five of seven participants) and in the Talca focus group (five of seven participants), whereas the Santiago focus group included no participants identifying as women and was instead composed of cisgender men together with non-binary participants whose discourse circulated more fluidly across gender categories. Two distinct but related issues follow. The first concerns access: the recruitment networks available to the team in each site likely shaped which participants were reached, and the absence of women in the Santiago focus group must be acknowledged as a constraint on the comparative reading of that site. The second issue is more substantive, and concerns how the predominant gendered registers of each encounter shaped the discursive coordinates of the conversation. In encounters where masculinities (both cis-masculine and non-binary-masculine) predominated—most clearly in the Santiago focus group—the discussion gravitated toward productivity, work, money, class, social mobility, and aesthetic codes of recognition, while gender identity itself was articulated as comparatively fluid. In encounters where femininities predominated—most clearly in the Talca focus group and in the individual interviews—the discussion tended to be organized around care, the texture of intimate bonds, and the work of sustaining ties under conditions of surveillance. These contrasts are productive analytically, in that they suggest that what emerges as “the territorial” in each site is not independent of the gendered subject-positions that animate the conversation. At the same time, they constitute a limitation, because the comparative reading between Talca and Santiago rests on encounters whose gender composition was asymmetric. A future iteration of this comparative design should aim for a more balanced gender composition across sites and should explicitly thematize how the gendered registers of each encounter modulate the territorial narratives that emerge.

## 6. Conclusions

Within the scope of a young, predominantly cisgender, university-educated sample, this study contributes qualitative evidence that territorial scale operates as a constitutive condition, not merely contextual, of LGBTIQA+ violence and survival in Talca and Santiago. The statement “Talca is not Santiago” condenses a moral geography where differential anonymity, network density, social surveillance, and access to information configure distinct thresholds of visibility, risk, and agency. The notion of “infrastructure of possibility” allows us to name the territorial assemblage of resources that makes LGBTIQA+ life practicable, or precarizes it, at a given scale, and to avoid readings that privatize care as individual responsibility.

The findings also warn against simplistic idealizations: the metropolis does not guarantee universal freedom, and the medium-sized city or rurality does not determine total closure. Participants’ experiences show gradients, nuances, and counterexamples that invite territorially sensitive analyses. The study also underscores that interventions oriented to reducing LGBTIQA+ violence require attention to the material and relational conditions that make it possible, or impossible, to seek support, recompose networks, and sustain a dignified life in concrete territories.

As discussed in Section 5.4, the findings suggest several directions for future research. First, studies including trans women, older adults, and LGBTIQA+ persons without university education in medium-sized cities and ruralities are essential to address experiences that this corpus cannot speak to. Second, comparative studies incorporating other Chilean medium-sized cities (Temuco, Valdivia, Antofagasta) would allow us to evaluate whether the identified patterns are replicated or vary according to specific regional configurations. Third, research examining violence in digital spaces, an emerging theme in the focus groups but not developed in this analysis, could complement the understanding of contemporary infrastructures of possibility, particularly for young LGBTIQA+ people. Fourth, longitudinal studies could trace how mobility trajectories effectively reconfigure survival possibilities over time. Fifth, research with an intersectional approach that more explicitly incorporates dimensions of class, racialization, and disability would help to complexify the understanding of how territory is articulated with other axes of inequality.

From an applied perspective, the results show that intervening in LGBTIQA+ violence and survival requires attention to territorial conditions: connectivity, information circulation, recognizable support routes, community spaces, and confidentiality safeguards appropriate to medium-sized cities and ruralities. This is consistent with Ahmed’s [18] warning about the distance between institutional discourses of diversity and effective material conditions, and with recent critiques of neoliberal affects in corporate diversity discourses [83]. In contexts of high social surveillance, strengthening “infrastructures” means designing supports that do not expose those who seek help, and recognizing that violence can also operate within community networks, so that devices must contemplate network recomposition, discreet accompaniments, and alternative circuits.

## Supporting information

S1 Checklist COREQ checklist. Completed Consolidated Criteria for Reporting Qualitative Research (COREQ) 32-item checklist, with an item-by-item indication of where each item is reported in the manuscript.
(DOCX)

S1 File Analytic codebook. Codebook documenting 27 codes organized across the three themes (9 codes per theme), with code identifiers, definitions, inclusion and exclusion criteria, and an anonymized exemplar for each code, together with discarded codes and their rationale, reflexive notes from team debriefings, and a glossary of regional and Chilean Spanish terms. The codebook is deposited in the Zenodo public repository under a Creative Commons Attribution 4.0 International (CC BY 4.0) license, with the citable Digital Object Identifier https://doi.org/10.5281/zenodo.20171907.
(DOCX)
