# Supplementary material for: Talca no es Santiago: Moral geography, rurality, and infrastructure of possibility in LGBTIQA+ violence and survival
Source: PLoS One. 2026 Oct 14;21(10):e0360622. doi: 10.1371/journal.pone.0360622 (PMC13667764; doi:10.1371/journal.pone.0360622)
Supplement: Supplementary file 1 [file pone.0360622.s001.docx]

**S1 Checklist. COREQ Checklist**

**Consolidated Criteria for Reporting Qualitative Research (COREQ): 32-item checklist**

Tong A, Sainsbury P, Craig J (2007). International Journal for Quality in Health Care, 19(6), 349–357. doi:10.1093/intqhc/mzm042

**Manuscript:** Talca no es Santiago: Moral geography, rurality, and infrastructure of possibility in LGBTIQA+ violence and survival (PONE-D-26-02900, second revision)

**Authors:** Wilson Orlando Albornoz Fuentes (Universidad Autónoma de Chile, Talca, Chile), Cecilia Valenzuela (Universidad Autónoma de Chile, Talca, Chile), Thiare Ramírez (Universidad Autónoma de Chile, Talca, Chile)

The following checklist documents how each of the 32 COREQ items is addressed in the revised manuscript. Section references correspond to the labeled sections in the revised manuscript file (Manuscript_Revised_TrackedRed.docx).

| **No.** | **Item** | **Guide question/description** | **Reported in the manuscript** |
| --- | --- | --- | --- |
| **Domain 1: Research team and reflexivity** | | | |
| **Personal characteristics** | | | |
| 1 | **Interviewer/facilitator** | Which author(s) conducted the interview or focus group? | The principal investigator (Wilson Albornoz, WOAF) conducted all individual interviews and facilitated both focus groups. Co-researchers Cecilia Valenzuela (CV) and Thiare Ramírez (TR) participated in fieldwork support and post-session debriefings (see Sections 3.2 and 3.6). |
| 2 | **Credentials** | What were the researcher's credentials? E.g., PhD, MD | PI: clinical psychologist with doctoral training, affiliated with Universidad Autónoma de Chile (Talca, Chile). CV and TR: psychologists in training based at Universidad Autónoma de Chile, Talca, Chile (Section 3.2, paragraphs 1–2). |
| 3 | **Occupation** | What was their occupation at the time of the study? | The PI is a researcher affiliated with Universidad Autónoma de Chile (Talca, Chile), where the Fondecyt project is hosted; CV and TR are researchers at Universidad Autónoma de Chile (Talca campus). The PI conducts a Fondecyt-funded research line on LGBTIQA+ corporalities and violence (Title block; Section 3.2). |
| 4 | **Gender** | Was the researcher male or female? | PI self-identifies as a homosexual man. CV and TR self-identify within sexual or gender minorities (Section 3.2, paragraphs 1–2). |
| 5 | **Experience and training** | What experience or training did the researcher have? | PI: ten years of experience in qualitative research on corporalities and LGBTIQA+ violence processes. CV and TR: experience in qualitative analysis applied to gender and sexuality research (Section 3.2, paragraphs 1–2). |
| **Relationship with participants** | | | |
| 6 | **Relationship established** | Was a relationship established prior to study commencement? | No prior relationship was established with participants. The researcher's position as a member of the studied community was made explicit at the beginning of each encounter (Section 3.2, paragraph 1). |
| 7 | **Participant knowledge of the interviewer** | What did the participants know about the researcher? E.g., personal goals, reasons for doing the research | Participants were informed that the researcher self-identifies as a member of the LGBTIQA+ community, that the questions arose from an academic (not therapeutic) interest, and were briefed on the broader research project (Section 3.2, paragraph 1; Section 3.8 Ethical Considerations). |
| 8 | **Interviewer characteristics** | What characteristics were reported about the interviewer/facilitator? E.g., bias, assumptions, reasons and interests in the research topic | Researcher positionalities are explicitly reported: PI self-identifies as a homosexual man and resides in a rural area near Talca; CV and TR self-identify within sexual or gender minorities and have community-oriented experience in the Maule region. Positionalities are treated as analytical resources rather than sources of bias to be neutralized, consistent with Braun and Clarke's reflexive epistemology (Section 3.2, paragraphs 1–2). |
| **Domain 2: Study design** | | | |
| **Theoretical framework** | | | |
| 9 | **Methodological orientation and theory** | What methodological orientation was stated to underpin the study? E.g., grounded theory, discourse analysis, ethnography, phenomenology, content analysis | Reflexive Thematic Analysis (RTA) following Braun and Clarke (2006), grounded in critical geographies of sexuality, subjectivation theory, and Latin American LGBTIQA+ studies (Section 2 Theoretical framework; Section 3.1 Study Design). |
| **Participant selection** | | | |
| 10 | **Sampling** | How were participants selected? E.g., purposive, convenience, consecutive, snowball | Non-probabilistic sampling combining purposive sampling by criteria with snowball sampling from community networks (Section 3.4, paragraph 1). |
| 11 | **Method of approach** | How were participants approached? E.g., face-to-face, telephone, mail, email | Recruitment was articulated through two LGBTIQA+ collectives in Talca (one university-linked, one community-based, both with territorial base in the Maule region) and one organization with metropolitan presence in Santiago, complemented by dissemination on social networks. These organizations disseminated the call and, in some cases, mediated initial contact with potential participants (Section 3.5, paragraphs 1–2). |
| 12 | **Sample size** | How many participants were in the study? | Analytical subcorpus: 7 individual interviews + 2 focus groups (Talca FG, n=7; Santiago FG, n=5), drawn from a broader Fondecyt project (N° 11251504) currently comprising a total of 42 participants. The decision to conclude data collection with this corpus was guided by the information-power framework of Malterud, Siersma and Guassora (2016) (Sections 3.1, 3.4, 3.6). |
| 13 | **Non-participation** | How many people refused to participate or dropped out? Reasons? | No participant who provided informed consent dropped out of the analytical subcorpus. Some individuals contacted through community networks did not respond to invitations; specific reasons were not documented, as the study did not systematically record non-response (acknowledged in Section 5.4 Limitations as part of the discussion of recruitment selection effects). |
| **Setting** | | | |
| 14 | **Setting of data collection** | Where was the data collected? E.g., home, clinic, workplace | All individual interviews and focus groups were conducted in person, in private spaces appropriate to confidentiality (Section 3.6, paragraphs 1–2; Section 3.8 Ethical Considerations regarding anonymization and confidentiality). |
| 15 | **Presence of non-participants** | Was anyone else present besides the participants and researchers? | Individual interviews were conducted in dyadic format (PI and participant); focus groups included only the participants and the PI as facilitator. No non-participants were present during data collection (Section 3.6). |
| 16 | **Description of sample** | What are the important characteristics of the sample? E.g., demographic data, date | Detailed sociodemographic characteristics are reported in Tables 1, 2, and 3, including age, education, gender identity, sexual orientation, and place of origin / territorial trajectory. Recruitment took place between 02/07/2025 and 01/09/2025 (Tables 1–3; Section 3.5). |
| **Data collection** | | | |
| 17 | **Interview guide** | Were questions, prompts, guides provided by the authors? Was it pilot tested? | Semi-structured interview guide and focus-group prompts were developed by the team and iteratively refined during post-fieldwork debriefings. The instruments are available from the corresponding author upon reasonable request (Sections 3.6 and 3.2). |
| 18 | **Repeat interviews** | Were repeat interviews carried out? If yes, how many? | Yes. Participation included between one and three sessions per individual-interview participant, depending on the depth of the narrative and scheduling availability (Section 3.6, individual interviews paragraph). |
| 19 | **Audio/visual recording** | Did the research use audio or visual recording to collect the data? | All interviews and focus groups were audio-recorded with prior written authorization from participants and subsequently transcribed verbatim for analysis (Section 3.6; Section 3.8 Ethical Considerations). |
| 20 | **Field notes** | Were field notes made during and/or after the interview or focus group? | Yes. Field notes were taken by the principal investigator during and after each session and were jointly reviewed by the three team members during post-session debriefings (Section 3.6, team-based fieldwork paragraph). |
| 21 | **Duration** | What was the duration of the interviews or focus group? | Individual interviews: between 45 and 180 minutes per participant in total (across one to three sessions). Focus groups: approximately 2 hours and 30 minutes each (Section 3.6). |
| 22 | **Data saturation** | Was data saturation discussed? | Yes. Saturation was explicitly not used as a stopping criterion, as it is epistemologically inconsistent with the reflexive thematic analysis adopted here. The information-power framework of Malterud, Siersma and Guassora (2016) is invoked instead, mapping its five dimensions onto the present study (Section 3.4, paragraph 2). |
| 23 | **Transcripts returned** | Were transcripts returned to participants for comment and/or correction? | Transcripts were not returned to participants for review. This decision is consistent with the reflexive epistemology adopted: interpretive responsibility lies with the analytical team, who treated their positionalities as analytical resources rather than sources of bias to be neutralized through member checking (Section 3.7; Section 5.4 Limitations regarding qualitative interpretive design). |
| **Domain 3: Analysis and findings** | | | |
| **Data analysis** | | | |
| 24 | **Number of data coders** | How many data coders coded the data? | Three coders: the principal investigator (WOAF) and two co-researchers (CV, TR). All three participated in independent familiarization, initial code generation on a shared subset, consensus-building meetings, and collaborative application of the codebook to the rest of the corpus (Section 3.7, paragraph 2). |
| 25 | **Description of the coding tree** | Did authors provide a description of the coding tree? | A working codebook was developed through consensus meetings and applied collaboratively across the corpus. The three constructed themes and their constitutive analytical foci are described in detail in Section 4 (Results). The full codebook is publicly available as Supplementary File S2, deposited at the Zenodo repository under a Creative Commons Attribution 4.0 International (CC BY 4.0) license with the citable Digital Object Identifier https://doi.org/10.5281/zenodo.20171907. The deposited codebook documents 27 codes organized across the three themes (Theme 1: 9 codes; Theme 2: 9 codes; Theme 3: 9 codes), each with code ID, definition, inclusion and exclusion criteria, and an anonymized exemplar; it also documents discarded codes with their rationale, reflexive notes from team debriefings, and a glossary of regional and Chilean Spanish terms (Sections 3.7 and 4). |
| 26 | **Derivation of themes** | Were themes identified in advance or derived from the data? | Themes were constructed inductively from the data through the six phases of Reflexive Thematic Analysis (Braun and Clarke, 2006), in dialogue with the theoretical framework. They were not identified in advance (Section 3.7, paragraph 1). |
| 27 | **Software** | What software, if applicable, was used to manage the data? | Transcripts were managed manually by the team. No specialized qualitative analysis software (e.g., NVivo, ATLAS.ti) was used; coding and codebook development were conducted through iterative team meetings on annotated transcripts, consistent with the reflexive interpretive epistemology adopted (Section 3.7). |
| 28 | **Participant checking** | Did participants provide feedback on the findings? | Participants did not provide formal feedback on the constructed themes. As noted under item 23, this is consistent with the reflexive thematic analysis approach adopted, in which interpretive authority resides in the analytical team; the limitations of this design are acknowledged in Section 5.4. |
| **Reporting** | | | |
| 29 | **Quotations presented** | Were participant quotations presented to illustrate the themes/findings? Was each quotation identified? E.g., participant number | Yes. Throughout Section 4 (Results), each theme is illustrated with multiple anonymized verbatim quotations. Each quotation is identified with an alphanumeric code that preserves anonymity while indicating the data source (e.g., P-E-1 for individual interview 1; P-GF-T-3 for focus group Talca, participant 3; P-GF-S-2 for focus group Santiago, participant 2). The full coding key is given in Tables 1–3. |
| 30 | **Data and findings consistent** | Was there consistency between the data presented and the findings? | Yes. Each constructed theme in Section 4 is grounded in multiple quotations across interviews and both focus groups, and the Discussion (Section 5) returns systematically to the empirical material to anchor each theoretical claim. The triangulation process described in Section 3.7 was designed to ensure this consistency. |
| 31 | **Clarity of major themes** | Were major themes clearly presented in the findings? | Yes. Three major themes are explicitly named and developed: (5.1 / Theme 1) Infrastructure of possibility: information, networks, and spaces; (5.2 / Theme 2) Social surveillance and reputation: feeling observed, rumor, and daily control; (5.3 / Theme 3) Mobility and education as partial opening (Sections 4.1–4.3 in Results; Sections 5.1–5.3 in Discussion). |
| 32 | **Clarity of minor themes** | Is there a description of diverse cases or discussion of minor themes? | Yes. The analysis explicitly incorporates counterexamples and nuancing cases that complicate a linear urban/rural reading, for instance, a participant from a rural area near Talca (Purísima) whose extended family did not exercise sanction, and the intraregional contrast between Talca and Linares. These minor patterns are surfaced in Section 4 and recovered in the Discussion (Section 5) as part of the productive analytical tension the manuscript sustains. |
