# Supplementary material for: Talca no es Santiago: Moral geography, rurality, and infrastructure of possibility in LGBTIQA+ violence and survival
Source: PLoS One. 2026 Oct 14;21(10):e0360622. doi: 10.1371/journal.pone.0360622 (PMC13667764; doi:10.1371/journal.pone.0360622)
Supplement: Supplementary file 2 [file pone.0360622.s002.docx]

**Codebook**: Talca no es Santiago: Moral geography, rurality, and infrastructure of possibility in LGBTIQA+ violence and survival

Reflexive Thematic Analysis - Code Definitions and Analytic Procedure

Wilson Orlando Albornoz Fuentes

Cecilia Valenzuela

Thiare Ramírez

Fondecyt de Iniciación N° 11251504 (ANID, Chile)

Universidad Autónoma de Chile

Version 11.5

May 2026

License: Creative Commons Attribution 4.0 International (CC BY 4.0)

DOI: [to be assigned upon deposit]

# Contents

1. Purpose of this document

2. Recommended citation

3. Study context and ethical approval

4. Theoretical orientation

5. Analytic team and positionality

6. Analytic procedure

7. Principles for code generation and retirement

8. Master code list

8.1 Theme 1 - Infrastructure of possibility

8.2 Theme 2 - Surveillance and reputation

8.3 Theme 3 - Mobility and education as partial opening

9. Discarded codes and rationale

10. Reflexive notes from team debriefings

11. Glossary of regional and Chilean Spanish terms

12. License and reuse

# 1. Purpose of this document

This codebook documents the analytic process used to construct the three themes reported in the article "Talca no es Santiago: Moral geography, rurality, and infrastructure of possibility in LGBTIQA+ violence and survival." It is designed to be deposited in a public repository as a supporting material under an open license, in order to provide transparency about the analytic decisions taken without disclosing identifiable transcript content.

The codebook serves four explicit purposes:

(i) to make the analytic structure of the article auditable by other qualitative researchers;

(ii) to document the inductive grounding of theme construction by reporting both the codes that sustained themes and those that were retired during analysis;

(iii) to comply with the data-availability requirements of the journal in a manner that is ethically defensible given the confidentiality of transcript material from a hidden and vulnerable population in a medium-sized city;

(iv) to facilitate methodological reuse by other research teams working on LGBTIQA+ violence, territoriality, or moral geography in Latin American contexts.

This codebook is not a closed protocol. Codes are presented with definitions, inclusion and exclusion criteria, and a brief anonymized exemplar, but their meaning is consubstantial with the analytic process described in the article and with the reflexive epistemology of Braun and Clarke's Reflexive Thematic Analysis (RTA), which understands themes as situated interpretive constructions rather than as patterns waiting to be discovered.

# 2. Recommended citation

Albornoz Fuentes, W. O., Valenzuela, C., & Ramírez, T. (2026). Codebook for "Talca no es Santiago: Moral geography, rurality, and infrastructure of possibility in LGBTIQA+ violence and survival". Version 1.0. [Repository to be determined]. DOI: [to be assigned upon deposit].

# 3. Study context and ethical approval

The data analyzed in the parent article come from a subcorpus of a broader research project funded by Fondecyt de Iniciación N° 11251504 (ANID, Chile), titled "Heterocisnormative discourses and practices, and violence against feminized bodies in LGBTIQA+ relationships and contexts in Talca and Santiago."

The subcorpus consists of seven semi-structured individual interviews and two focus groups (Talca, n=7; Santiago, n=5), all conducted in person in the city of residence of each participant. Recruitment took place through LGBTIQA+ organizations and snowball referrals between February and September 2025.

All procedures were approved by the Ethics Committee of Universidad Autónoma de Chile (Protocol No. CEC 07-25, approved 20 March 2025). Written informed consent was obtained from all participants. Audio recordings, transcripts, and field notes are securely stored on encrypted devices and are not publicly shareable, in order to protect the confidentiality of participants from a hidden minority population in a medium-sized city where social legibility and reputational traceability create elevated reidentification risks. This codebook is the analytic-procedural record of the work, designed precisely so that the analytic process can be inspected publicly while the raw data remain protected.

# 4. Theoretical orientation

The analysis is anchored in critical geographies of sexuality and in Foucauldian-inflected accounts of subjectivation, with attention to debates on visibility, recognition, and stigma. The construction of themes takes territory not as a passive backdrop but as an active condition of habitability, distributing differential thresholds of anonymity, network density, information circulation, and the availability of LGBTIQA+ scenes. The notion of "infrastructure of possibility" is developed in the article as a way of naming the material-discursive assemblage of resources that makes LGBTIQA+ life practicable, or precarizes it, at a given urban scale. Visibility is treated as a relational and situated condition, not as an individual attribute; reputation is treated as a moral technology operating in dense relational networks; and mobility through education is treated as a partial rupture of territorial enclosure, articulated with class trajectories. The full theoretical apparatus and citations are developed in Sections 1, 2, and 5 of the article.

Importantly, although these theoretical commitments oriented the team's attention, the construction of themes was not derived top-down from theory. Section 7 of this codebook documents the inductive principles followed during code generation and the codes that were retired because the corpus did not sustain them.

# 5. Analytic team and positionality

The analytic team comprised three researchers: the principal investigator (Wilson Albornoz, PhD-trained clinical psychologist, ten years of qualitative research experience on LGBTIQA+ violence; self-identifies as a homosexual man; resident in a rural area near Talca), and two co-researchers (Cecilia Valenzuela and Thiare Ramírez, psychologists in training based at Universidad Autónoma de Chile, Talca campus; both self-identify within sexual or gender minorities and are familiar with the territorial contexts under study).

Beyond their analytical role, the two co-researchers also participated in the data-collection phase: fieldwork logistics (scheduling, contact with potential participants through community networks, focus-group venue coordination), transcription quality checks, and regular post-fieldwork team debriefings in which ongoing interviews and focus-group sessions were jointly reviewed. The team's shared situatedness in Talca grounded both data collection and analysis in lived knowledge of the region; individual interpretive emphases were systematically discussed in team meetings to maintain analytical rigor and reflexivity. Positionalities were treated as analytical resources rather than sources of bias to be neutralized, consistent with the reflexive epistemology of RTA.

# 6. Analytic procedure

The analysis followed the six phases of Reflexive Thematic Analysis (Braun and Clarke, 2006; 2019):

Phase 1 - Familiarization. Each team member read the full corpus of transcripts independently, taking initial impressions and memos.

Phase 2 - Generation of initial codes. On a shared subset of transcripts, the three team members generated initial codes independently. Codes were oriented to territorial references, experiences of surveillance and visibility, support networks, available spaces, information circuits, and mobility trajectories. A substantially broader set of codes than those finally reported was generated in this phase (see Section 9, Discarded codes).

Phase 3 - Search for themes. Codes were collated into candidate themes through consensus-building meetings, in which definitions were refined, redundancies merged, and a working codebook was developed.

Phase 4 - Review of themes. Candidate themes were checked against the coded extracts and against the full corpus, verifying internal coherence and external distinction. Codes that did not consolidate into robust patterns were retired, and content was iteratively reassigned where appropriate.

Phase 5 - Definition and naming of themes. The three themes finally reported were defined and named. Each theme was articulated around three to four subfoci, documented in the article's Results section.

Phase 6 - Report production. The article was drafted iteratively, with all three team members reviewing and revising.

Weekly team meetings during Phases 2-5 functioned as the principal site of triangulation. Inter-coder agreement metrics were not used, consistent with Braun and Clarke's reflexive epistemology, which treats divergence among coders as productive of interpretive depth rather than as noise to be minimized. Disagreements were resolved by returning to the transcripts and to the theoretical framework.

# 7. Principles for code generation and retirement

Three principles guided the analytic work:

Principle 1 - Inductive grounding. Codes were generated close to participants' formulations, often using in-vivo language. Theoretical concepts were used to organize and articulate themes, but a code was only retained if its presence in the corpus was dense enough and sufficiently articulated across cases to support a robust pattern of meaning.

Principle 2 - Retirement of overdetermined codes. Codes that initially appeared theoretically promising but whose density in the corpus was insufficient, or whose variations across cases were too heterogeneous to be consolidated under a single theme, were progressively retired from the analytic frame. Section 9 lists these codes and the rationale for their retirement.

Principle 3 - Reabsorption where appropriate. Content originally coded under retired categories was, where pertinent, reabsorbed into the three final themes. For example, references to economic dependency in intimate relationships, initially coded as economic violence, were reabsorbed into Theme 2 in connection with the discussion of the territorial conditions for leaving violent bonds; references to the university as an institutional space, initially coded as institutional access, were reabsorbed into Theme 3 as a partial opening through education.

# 8. Master code list

The codes are organized by theme. Each code entry includes: code ID, code name, definition, inclusion criteria, exclusion criteria, and one brief anonymized exemplar. Exemplars are paraphrastic indications of the kind of content captured by the code, not verbatim quotations from the transcripts. Verbatim quotations are reserved for the article itself and for the corresponding-author archive.

## 8.1 Theme 1 - Infrastructure of possibility: information, networks, and spaces

This theme groups codes that capture the differential availability, across territories, of the material and discursive resources that make LGBTIQA+ life practicable: information circuits, support references, physical spaces, language for naming the experience, and the family-school configurations that condition access to these resources.

| **Code ID** | **C1.1** |
| --- | --- |
| **Code name** | Access to information and language for naming the experience |
| **Definition** | Participants describe (the absence or presence of) the words, categories, and informational resources needed to name same-sex desire, gender non-conformity, or LGBTIQA+ identity during formative years and in adulthood. |
| **Inclusion criteria** | Statements where the participant describes not knowing how to name what they felt; references to discovering vocabulary at a specific age; mentions of media, conversations, or readings that provided language. |
| **Exclusion criteria** | Generic references to identity formation that do not include language, vocabulary, or informational access as the focus. |
| **Exemplar** | A participant raised in a rural-religious context describes not having the language to name same-sex attraction until adolescence, when fragmentary information from media became accessible. |

| **Code ID** | **C1.2** |
| --- | --- |
| **Code name** | Differential media connectivity and information circuits |
| **Definition** | Participants describe how access to LGBTIQA+ content, news, and references is mediated by territorial conditions of connectivity, available media, and physical isolation. |
| **Inclusion criteria** | References to specific media (radio, social media, streaming platforms); mentions of places where connectivity is poor or absent; comparisons between media availability in metropolitan and non-metropolitan contexts. |
| **Exclusion criteria** | References to digital surveillance or to traceability through social media (these are captured under C2.2). |
| **Exemplar** | A participant describes that in their rural area only AM radio was reliable and that LGBTIQA+ content never circulated through that medium. |

| **Code ID** | **C1.3** |
| --- | --- |
| **Code name** | **Presence or absence of formal support references** |
| **Definition** | **Participants name (or report not knowing) formal organizations, services, helplines, or referral points for LGBTIQA+ persons.** |
| **Inclusion criteria** | **Mentions of specific organizations by name; statements about not knowing any support network locally; comparisons between the availability of references in different cities.** |
| **Exclusion criteria** | **References to informal community networks, which are captured under C1.4.** |
| **Exemplar** | **A participant in a medium-sized city states they are aware of organizations in the capital but do not know any local equivalent.** |

| Code ID | C1.4 |
| --- | --- |
| Code name | **Self-organized community practices and informal coordination** |
| Definition | Participants describe LGBTIQA+ networks, peer groups, and informal coordination practices that emerge in the absence of robust formal infrastructure. |
| Inclusion criteria | References to peer-led groups, informal gatherings, WhatsApp groups, and other locally generated networks; statements about mutual support practices among community members. |
| Exclusion criteria | Mentions of formal organizations (captured under C1.3); references to digital coordination as surveillance risk (captured under C2.2). |
| Exemplar | A participant describes a locally organized WhatsApp group through which community members coordinate gatherings and share information. |

| **Code ID** | **C1.5** |
| --- | --- |
| **Code name** | Availability of physical LGBTIQA+ spaces |
| **Definition** | Participants describe the existence (or scarcity) of bars, cultural venues, or community spaces where LGBTIQA+ persons can gather. |
| **Inclusion criteria** | Mentions of specific spaces; comparisons between cities in terms of available scenes; statements about traveling to access spaces; descriptions of internal stratification within spaces. |
| **Exclusion criteria** | References to private homes or domestic spaces; references to digital spaces only. |
| **Exemplar** | A participant describes visiting a lesbian bar in the capital and the affective impact of seeing lesbians of different ages and occupations. |

| **Code ID** | **C1.6** |
| --- | --- |
| **Code name** | Generational continuity and visibility of LGBTIQA+ trajectories |
| **Definition** | Participants describe the importance of seeing or not seeing older LGBTIQA+ persons living visibly, as a condition for imagining biographical continuity. |
| **Inclusion criteria** | Statements about not seeing older LGBTIQA+ persons in their context; descriptions of encounters with older role models; reflections on the foreclosure of future imagination in the absence of such figures. |
| **Exclusion criteria** | Generic references to age that do not connect to identity visibility or biographical horizon. |
| **Exemplar** | A participant comments that seeing older lesbians in a bar in the capital made tangible the possibility of a queer life beyond youth. |

| **Code ID** | **C1.7** |
| --- | --- |
| **Code name** | Religious schooling and the foreclosure of language |
| **Definition** | Participants describe how Catholic or otherwise religious schooling foreclosed the possibility of naming or discussing sexual and gender diversity during formative years. |
| **Inclusion criteria** | Mentions of attending Catholic or religious schools; descriptions of explicit prohibitions, expulsions, or conversion therapy referrals; reports of the topic being unspeakable. |
| **Exclusion criteria** | References to religious family environments that are not about schooling (captured under C1.8). |
| **Exemplar** | A participant describes that at their Catholic high school, openly lesbian students were expelled without formal explanation. |

| **Code ID** | **C1.8** |
| --- | --- |
| **Code name** | Family-imposed restrictions on access |
| **Definition** | Participants describe family-imposed limits on attending events, accessing community spaces, or expressing identity, motivated by fear of sanction (including physical violence). |
| **Inclusion criteria** | Reports of being forbidden to attend cultural events; statements about hiding identity to avoid physical violence at home; descriptions of explicit family prohibitions. |
| **Exclusion criteria** | Family configurations that operate as ambivalent support (captured under C1.9). |
| **Exemplar** | A participant recalls being unable to attend cultural events due to family prohibitions motivated by fear of paternal physical violence. |

| **Code ID** | **C1.9** |
| --- | --- |
| **Code name** | Rural family configurations as ambivalent support |
| **Definition** | Participants describe rural family contexts that, contrary to the general expectation of restriction, operated as supportive environments through specific intergenerational dynamics. |
| **Inclusion criteria** | Reports of older rural relatives expressing acceptance or worry-as-care; statements that complicate a linear association between rurality and restriction; descriptions of intergenerational warmth that exceeds normative expectations. |
| **Exclusion criteria** | Urban family acceptance; rural contexts described as uniformly restrictive. |
| **Exemplar** | A participant from a rural sector describes that their grandfather, in his seventies, responded with concern for their safety rather than with rejection of their sexual orientation. |

## 8.2 Theme 2 - Surveillance and reputation: feeling observed, rumor, and daily control

This theme groups codes that capture how social legibility intensifies in medium-sized cities and rural environments, producing forms of surveillance, reputational damage, and shame that restrict the margin for manoeuvre, including the conditions for asking for help without activating secondary sanctions.

| **Code ID** | **C2.1** |
| --- | --- |
| **Code name** | Sensation of being watched and public hyperlegibility |
| **Definition** | Participants describe the everyday experience of feeling observed or read by others in public space, and the calculations of self-conduct that follow from that experience. |
| **Inclusion criteria** | Statements about feeling watched in specific places; reports of restricting affective gestures (hand-holding, kissing) due to perceived surveillance; descriptions of the city as a place where 'everyone knows everyone'. |
| **Exclusion criteria** | Generic references to discrimination that do not involve the affective dimension of being watched. |
| **Exemplar** | A participant describes restraining public displays of affection in a medium-sized city because of the constant sensation of being observed and judged. |

| **Code ID** | **C2.2** |
| --- | --- |
| **Code name** | Reputational traceability in dense networks |
| **Definition** | Participants describe how, in dense relational networks (medium-sized cities, small towns, professional milieus), information about one's intimate life is rapidly traceable through chains of acquaintances. |
| **Inclusion criteria** | References to 'small town, big hell'; descriptions of how news travels quickly through networks; reports of being recognized as 'X's child' or 'from such-and-such school'. |
| **Exclusion criteria** | Generic urban anonymity references; digital traceability through algorithms. |
| **Exemplar** | A participant describes that in their small home town everyone identifies others through family lineage, schools attended, and known associations. |

| **Code ID** | **C2.3** |
| --- | --- |
| **Code name** | Rumor as moral technology |
| **Definition** | Participants describe how rumor, gossip, or 'things they make up' operate as social mechanisms that regulate conduct and produce reputational damage. |
| **Inclusion criteria** | Reports of rumors circulated about the participant or about others in their community; descriptions of how rumors discourage participation in local LGBTIQA+ scenes; statements about avoiding spaces due to fear of becoming the subject of gossip. |
| **Exclusion criteria** | Direct verbal aggression (captured under C2.4 or C2.7). |
| **Exemplar** | A participant withdraws from local LGBTIQA+ spaces after rumors about them have circulated, indicating the costs of community participation in narrow networks. |

| **Code ID** | **C2.4** |
| --- | --- |
| **Code name** | Taboo and restriction of public affection |
| **Definition** | Participants describe explicit or implicit prohibitions on public expressions of affection between same-sex partners. |
| **Inclusion criteria** | Statements that affection is 'not allowed' or remains 'taboo' in specific places; reports of hostile reactions to public affection; descriptions of self-restraint motivated by these prohibitions. |
| **Exclusion criteria** | Family restrictions on identity expression at home (captured under C1.8). |
| **Exemplar** | A participant describes that public displays of affection with a same-sex partner are unthinkable in a smaller intra-regional city. |

| **Code ID** | **C2.5** |
| --- | --- |
| **Code name** | Intra-regional gradients of moral regulation |
| **Definition** | Participants articulate moral-territorial differences within the same region (for example, between a regional capital and smaller cities or rural localities) that exceed the urban/rural binary. |
| **Inclusion criteria** | Explicit comparisons between two non-metropolitan localities; descriptions of moral climate as a gradient rather than as a binary; references to specific cities or towns being more or less restrictive than others. |
| **Exclusion criteria** | Talca/Santiago comparisons (these structure the analysis as a whole and are not coded separately). |
| **Exemplar** | A participant contrasts Talca with a smaller nearby city, indicating that the latter remains a place where the topic is taboo and affective expression is explicitly forbidden. |

| **Code ID** | **C2.6** |
| --- | --- |
| **Code name** | Hegemonic masculinity as familial correction device |
| **Definition** | Participants describe how hegemonic masculinity operates within the family as a corrective mechanism, regulating gestures, behaviors, and household roles. |
| **Inclusion criteria** | Reports of explicit injunctions on how to move, speak, or perform domestic tasks; descriptions of paternal or fraternal correction motivated by perceived gender deviation; statements about constant policing of comportment. |
| **Exclusion criteria** | Family violence not related to gender comportment. |
| **Exemplar** | A participant recounts persistent paternal corrections on movement, speech, and domestic chores, framed as enforcement of acceptable masculinity. |

| **Code ID** | **C2.7** |
| --- | --- |
| **Code name** | Institutional violence in professional and educational contexts |
| **Definition** | Participants describe direct or indirect aggression, exclusion, or attempts to expel them from internships, workplaces, or educational institutions because of their sexual orientation or gender identity. |
| **Inclusion criteria** | Reports of explicit hostile statements by supervisors or teachers; descriptions of efforts to remove the participant from an institutional space; statements about institutional non-response to complaints. |
| **Exclusion criteria** | Family violence (captured under C2.6 or C1.8); rumor-based harm (captured under C2.3). |
| **Exemplar** | A participant describes a clinical-internship supervisor who openly stated that 'people like you' do not belong, and acted to have them removed. |

| **Code ID** | **C2.8** |
| --- | --- |
| **Code name** | Misgendering and deadnaming as intracommunity weapons |
| **Definition** | Participants describe how misgendering and deadnaming operate as deliberate weapons within families and within the LGBTIQA+ community itself, particularly during conflicts. |
| **Inclusion criteria** | Reports of family members using a deadname specifically during conflicts; descriptions of LGBTIQA+ peers using misgendering as retaliation; statements about the affective intensity of these acts. |
| **Exclusion criteria** | Misgendering experienced as ignorance rather than as weapon. |
| **Exemplar** | A non-binary participant describes that family members deploy their deadname precisely during arguments, indicating the act's instrumental character. |

| **Code ID** | **C2.9** |
| --- | --- |
| **Code name** | Shame and self-regulation |
| **Definition** | Participants describe internalized shame or self-regulation as enduring effects of surveillance and corrective practices. |
| **Inclusion criteria** | Statements about hiding oneself; descriptions of self-censorship as a sustained way of being; references to feeling small, exposed, or invalidated. |
| **Exclusion criteria** | Acute affective responses to specific events without enduring effect. |
| **Exemplar** | A participant describes prolonged self-concealment as a strategy for managing anticipated sanction in their formative environment. |

## 8.3 Theme 3 - Mobility and education as partial opening

This theme groups codes that capture how movement between territories, especially associated with university studies, enables encounters, expands repertoires, and reconfigures future horizons, while also recognizing that such openings are partial, reversible, and mediated by class and educational position.

| **Code ID** | **C3.1** |
| --- | --- |
| **Code name** | Mobility for university studies as partial territorial rupture |
| **Definition** | Participants describe relocations driven by university enrollment that produced shifts in available networks, vocabularies, and spaces. |
| **Inclusion criteria** | References to relocating for studies; statements connecting the move with new possibilities; descriptions of changes in family or peer configurations after the move. |
| **Exclusion criteria** | Mobility not motivated by studies; relocations described as failed or merely lateral. |
| **Exemplar** | A participant describes how moving to a regional capital for university opened new vocabularies and peer configurations that were unavailable in their hometown. |

| **Code ID** | **C3.2** |
| --- | --- |
| **Code name** | Intra-regional migration as biographical opening |
| **Definition** | Participants describe migrations within the same region (rural to medium-sized city; smaller city to regional capital) that produced biographical openings less dramatic than metropolitan migration but analytically distinct. |
| **Inclusion criteria** | Statements about relocating within the region; comparisons between place of origin and current city of residence within the same macro-region; descriptions of intra-regional contrasts. |
| **Exclusion criteria** | Migration to the metropolis (captured under C3.3 where it enables specific events). |
| **Exemplar** | A participant describes how moving from a smaller intra-regional town to the regional capital substantially expanded their margin for affective expression and peer encounter. |

| **Code ID** | **C3.3** |
| --- | --- |
| **Code name** | Specialized circuits accessed through travel to the metropolis |
| **Definition** | Participants describe travel to the metropolis to access specific community events, encounters, or scenes unavailable in their territory of residence. |
| **Inclusion criteria** | References to specific events attended in the capital; statements about the affective intensity of these encounters; descriptions of returning to the territory of residence after the event. |
| **Exclusion criteria** | Permanent migration to the metropolis. |
| **Exemplar** | A participant describes a transmasculine encounter held in the capital as an almost utopian experience that contrasted sharply with the available circuits in their city of residence. |

| **Code ID** | **C3.4** |
| --- | --- |
| **Code name** | University as relatively free space |
| **Definition** | Participants describe the university as a space of relative freedom compared with previous educational contexts (typically Catholic or otherwise religious secondary schools). |
| **Inclusion criteria** | Statements contrasting university and prior schooling; references to being able to express identity at university; descriptions of university as a turning point. |
| **Exclusion criteria** | Institutional violence at university (captured under C2.7); university as social-class marker (captured under C3.8). |
| **Exemplar** | A participant describes university as the first space in which expressing sexual orientation did not entail the threat of sanction faced at their religious secondary school. |

| **Code ID** | **C3.5** |
| --- | --- |
| **Code name** | Digital informal coordination through messaging applications |
| **Definition** | Participants describe the use of messaging applications, particularly WhatsApp, as everyday infrastructure for coordinating LGBTIQA+ gatherings, sharing information, and sustaining peer support. |
| **Inclusion criteria** | References to specific messaging-based groups; descriptions of how digital coordination compensates for the scarcity of formal infrastructure; mentions of the role of these channels in connecting peers. |
| **Exclusion criteria** | Digital traceability as risk (captured under C2.2); social media as identity expression. |
| **Exemplar** | A participant describes a WhatsApp group of LGBTIQA+ peers in a medium-sized city through which gatherings and informational exchange are coordinated. |

| **Code ID** | **C3.6** |
| --- | --- |
| **Code name** | Educational access as tool for familial transformation |
| **Definition** | Participants describe how the access of one or more family members to higher education set in motion shifts in the entire family's repertoires, vocabularies, and ways of engaging with diversity. |
| **Inclusion criteria** | Statements that name education as a tool that changed the family; reports of older relatives shifting their views after younger members entered university; descriptions of intergenerational transformation. |
| **Exclusion criteria** | Personal educational trajectories that do not address family-level effects. |
| **Exemplar** | A participant from a poor rural-rooted family describes how the entry of several members of their generation into university opened new conversations and acceptance dynamics across the wider family. |

| **Code ID** | **C3.7** |
| --- | --- |
| **Code name** | Rural-urban trajectories |
| **Definition** | Participants describe biographical trajectories that move between rural origins and urban current residence, framed as gradual processes rather than as discrete migrations. |
| **Inclusion criteria** | Descriptions of gradual family migration from rural to urban areas; statements about maintaining rural ties from an urban location; references to multi-generational mobility. |
| **Exclusion criteria** | Static rural or static urban references. |
| **Exemplar** | A participant describes how their extended family migrated from a rural locality to the regional capital across generations, prompted by successive members' access to schooling. |

| **Code ID** | **C3.8** |
| --- | --- |
| **Code name** | Class and educational position as mediators of openness |
| **Definition** | Participants articulate how class background and educational position mediate the access to and quality of the infrastructure of possibility described in the previous codes. |
| **Inclusion criteria** | Statements connecting class background to access to opportunities; reflections on the visibility differential between LGBTIQA+ persons with and without resources; descriptions of class-based stratification within LGBTIQA+ spaces themselves. |
| **Exclusion criteria** | Class references that do not bear on access to LGBTIQA+ resources or recognition. |
| **Exemplar** | A participant articulates that visibility within LGBTIQA+ activist circles is unevenly distributed and tends to concentrate around those with greater socioeconomic and educational resources. |

| **Code ID** | **C3.9** |
| --- | --- |
| **Code name** | Future horizon: imagining biographical continuity |
| **Definition** | Participants describe shifts in the imaginability of a LGBTIQA+ future as a consequence of mobility and educational access. |
| **Inclusion criteria** | Statements about being able or unable to imagine a future as an LGBTIQA+ person; references to the affective texture of encountering older LGBTIQA+ models (overlaps with C1.6 but focused on the horizon dimension); descriptions of expanded biographical horizons. |
| **Exclusion criteria** | Concrete reports of present life without explicit horizon dimension. |
| **Exemplar** | A participant describes that encountering older lesbian women in the metropolis enabled them to imagine, for the first time, a long-term lesbian life. |

# 9. Discarded codes and rationale

Consistent with Principle 2 in Section 7, the analysis began with a broader set of codes than those finally retained. The codes listed below were generated during early phases of coding but were progressively retired because their density in the corpus was insufficient to sustain a robust pattern of meaning, or because their variations across cases were too heterogeneous to consolidate under a single theme. Where appropriate, content originally coded under these categories was reabsorbed into the three final themes (Principle 3, Section 7). The retirement of these codes does not imply that the underlying phenomena are absent or unimportant; it indicates that within this specific subcorpus they could not be articulated as a robust theme.

### D1 - Economic violence within intimate relationships

Initially coded as a stand-alone category capturing economic control, withholding of money, or financial dependency as a mechanism of partner violence. Retired because, although the phenomenon appeared in several accounts, its territorial articulation was uneven and its specific dynamics differed sharply across cases (cohabiting versus non-cohabiting couples; income asymmetry; family financial entanglement). Content was reabsorbed into Theme 2 in connection with the territorial conditions for leaving violent bonds, where economic dependency intersects with reputational traceability and scarcity of alternative circuits.

### D2 - Cultural and political dynamics at the institutional / national scale

Initially coded as a category capturing references to elections, party politics, national legislation (e.g., equal-marriage law), and macro-level conservative/progressive cycles. Retired because, although these references appeared in the corpus, their territorial specificity was limited and they did not produce a pattern of meaning that differentiated Talca from Santiago in ways relevant to the article's argument. Content remains potentially useful for a future article focused on the political conjuncture and is preserved in analytic memos but is not part of the present manuscript.

### D3 - Access to institutional spaces: university as a setting

Initially coded as an institutional-access category capturing the university as a physical and bureaucratic space, including admissions, scholarship access, and bureaucratic recognition of gender identity. Retired as a stand-alone code because its institutional-bureaucratic dimensions were too heterogeneous to consolidate. Content was reabsorbed into Theme 3 (codes C3.1, C3.4, C3.6, C3.8), where the university appears as a relatively free space, as a tool of familial transformation, and as a marker of class-mediated openness.

### D4 - Workplace access and labor inclusion

Initially coded as a category capturing the workplace as a site of inclusion or exclusion for LGBTIQA+ persons, including hiring discrimination, workplace climate, and labor precariousness. Retired as a stand-alone code because workplace references in the subcorpus were uneven and tended to be subsumed under broader concerns about institutional violence (C2.7) or about productivity-oriented discursive registers in the focus-group dynamics. Workplace dynamics remain analytically open for future articles.

# 10. Reflexive notes from team debriefings

This section documents three reflexive observations that emerged from team debriefings during data collection and analysis. These are not formal findings; they are recorded here to make explicit the interpretive choices that shaped the construction of themes.

### 10.1 Gender composition of each encounter modulated discursive registers

The team observed during debriefings that the predominant gendered composition of each encounter shaped the discursive coordinates of the conversation. In encounters where masculinities (both cis-masculine and non-binary-masculine) predominated - most clearly in the Santiago focus group, the conversation gravitated toward productivity, work, money, class, social mobility, and aesthetic codes of recognition, while gender identity itself was articulated as comparatively fluid. In encounters where femininities predominated - most clearly in the Talca focus group and in the individual interviews - the conversation tended to be organized around care, the texture of intimate bonds, and the work of sustaining ties under conditions of surveillance. This observation informed the framing of Section 5.4 of the article as a limitation that is also analytically productive.

### 10.2 The team's situatedness in Talca shaped attention

The team's shared territorial situatedness in Talca produced a heightened sensitivity to the medium-sized city as analytical object, and an attentiveness to intraregional gradients (Talca/Linares, Talca/Chanco, Talca/Constitución, Talca/Curicó) that might have been less foregrounded by a metropolitan-anchored team. This was a productive interpretive position, but it also implies that the Santiago material was read partly through Talca-grounded analytical questions, rather than as a self-standing site. The article's framing of Santiago as a contrast site, rather than as a co-primary site, reflects this positionality.

### 10.3 Confidentiality constraints in a medium-sized city shaped the level of detail reportable

Throughout the analysis, the team worked under the awareness that, in a medium-sized city, the combination of profession, age, neighborhood, and specific biographical detail can quickly become reidentifying. This shaped decisions about which extracts to use in the article, how to anonymize them, and what level of detail to include in this codebook. The exemplars in Section 8 are deliberately paraphrastic and stripped of identifying particulars for this reason.

# 11. Glossary of regional and Chilean Spanish terms

Several terms used by participants and reproduced in the article carry meanings specific to the Chilean and southern-Chilean context. The following glossary clarifies the most relevant ones for non-Chilean readers.

| **Talca** | Regional capital of the Maule Region in central Chile, with a population of approximately 240,000. Functions in this study as the prototypical medium-sized city. |
| --- | --- |
| **Maule Region** | Administrative region of central Chile, predominantly agricultural, with Talca as its capital. The region includes the cities of Curicó, Linares, Constitución, and the rural localities of Chanco, Empedrado, Purísima, Lontué, Colbún, and Parrales referenced by participants. |
| **Comuna** | Administrative subdivision of a Chilean region; roughly equivalent to a municipality or borough. In metropolitan Santiago, comunas differ substantially in socioeconomic profile (e.g., Providencia vs. Maipú vs. Lampa). |
| **Pueblo chico, infierno grande** | Common Chilean expression literally translatable as 'small town, big hell', used to evoke the intensified visibility and rapid circulation of gossip in small relational networks. |
| **Cola** | Chilean colloquial term for a gay man, often appropriated from a pejorative origin. In several participants' speech it is used reflexively or affectively, not pejoratively. |
| **Camiona** | Chilean colloquial term for a masculine-presenting lesbian, often with a pejorative valence in heteronormative usage and an appropriative valence within community usage. |
| **Plumofobia** | Spanish-language term referring to hostility, within gay communities, toward  gay men who perform femininity (literally, 'feather-phobia'). |
| **Movilh** | Movimiento de Integración y Liberación Homosexual, a long-standing Chilean LGBTIQA+ organization with strong metropolitan presence. Mentioned by participants as a reference point that is recognized in Santiago but not in Talca. |
| **Salir del closet** | Spanish translation of 'to come out of the closet'. Used by participants both as an act and as an ongoing relational process. |
| **Chupallas** | Traditional rural hat worn in central-southern Chile, used here metonymically by a participant to evoke the conservative-agrarian political imaginary of the Maule Region. |

# 12. License and reuse

This codebook is released under the Creative Commons Attribution 4.0 International License (CC BY 4.0). Users are free to share and adapt the material for any purpose, including commercial use, provided that they give appropriate credit to the authors, provide a link to the license, and indicate if changes were made. The full license text is available at https://creativecommons.org/licenses/by/4.0/.

To cite this codebook, please use the citation provided in Section 2 of this document.

Inquiries about the underlying corpus (interview transcripts, focus-group transcripts, audio recordings, field notes) should be directed to the corresponding author. Access to the underlying corpus is restricted in order to protect the confidentiality of participants and remains subject to review by the Ethics Committee of Universidad Autónoma de Chile (Protocol CEC 07-25). Anonymized excerpts and analytic memos may be made available upon reasonable request, subject to a data-use and confidentiality agreement.
